# One-Pot Synthesis of Terminal Alkynes from Alkenes

**DOI:** 10.1021/jacsau.4c00599

**Published:** 2024-08-05

**Authors:** Cristina Bilanin, Amravati S. Singh, Lluis Martínez-Belenguer, Antonio Leyva-Pérez

**Affiliations:** Instituto de Tecnología Química (UPV-CSIC), Universitat Politècnica de València-Agencia Estatal Consejo Superior de Investigaciones Científicas, Avda. de los Naranjos s/n, 46022 Valencia, Spain

**Keywords:** alkynes, alkenes, one-pot reaction, dehydrogenative hydrosilylation, vinyl silane, iodosobenzene, ruthenium catalysis, acetylene

## Abstract

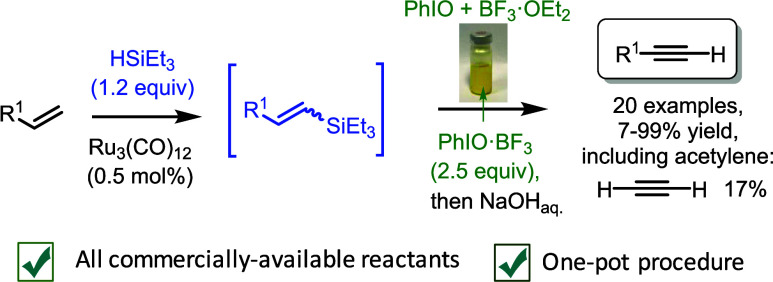

The direct synthesis of terminal alkynes from widely available terminal alkenes is an unmet challenge in organic synthesis. Here, we show that alkyl and aromatic terminal alkenes can be converted to the corresponding alkynes in a one-pot process consisting of a Ru-catalyzed dehydrogenative hydrosilylation, followed by an oxidative dehydrogenative reaction of the vinyl silane intermediate, enabled by the combination of PhIO with BF_3_. This formal alkene dehydrogenation reaction with commercially available reagents and under mild reaction conditions gives access to terminal alkynes in a simple manner, including acetylene.

## Introduction

**Figure 1:**
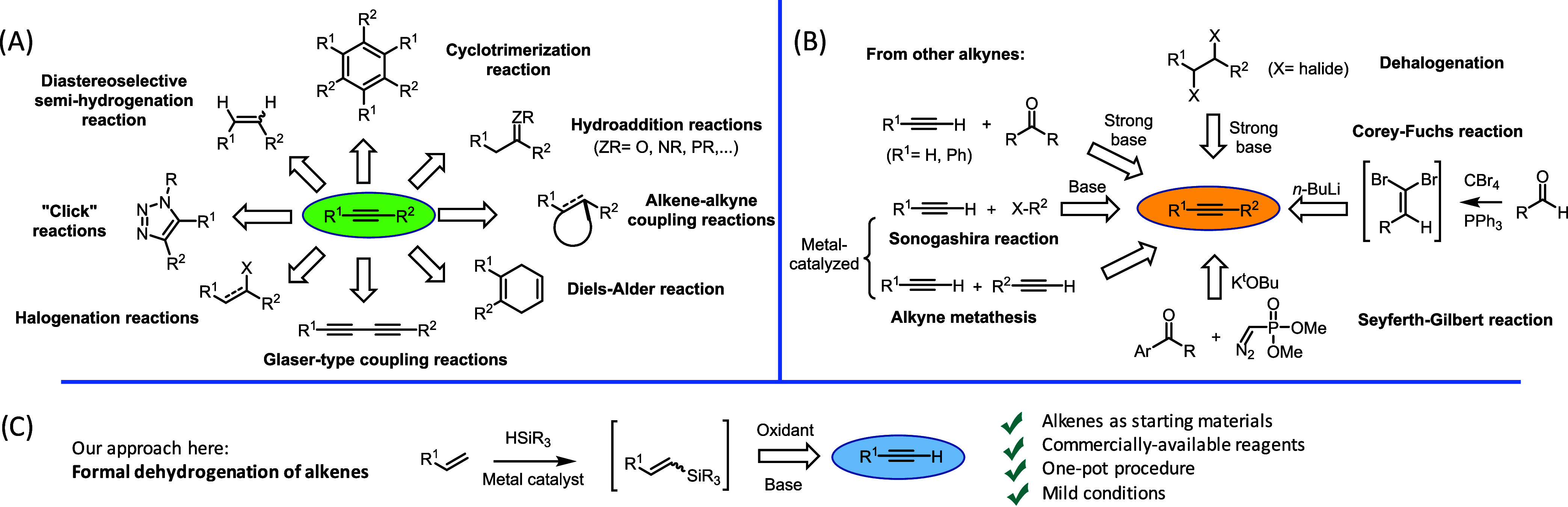
(A) Representative transformations of alkynes. (B) Main synthetic methods for alkynes. (C) The approach is proposed here.

Alkynes are pluripotential synthons in organic chemistry, transformable to a myriad of new compounds.^1^ The reactivity of alkynes not only arises from the energy released after breaking the C_sp_≡C_sp_ bond but also is enhanced by catalytic alkynophilic metals (Au, Pt, Cu, etc.), triggering a variety of metal-catalyzed reactions that have been widely studied during the last two decades (Figure 1A).^2^ Besides that, the relevance of alkynes in biological processes is gaining weight with the years: more and more naturally occurring alkynes are found, some of them with industrial use,^3^ and alkynes are the basis of the recently Nobel-awarded biorthogonal chemistry.^4^

In striking contrast to the recurrent use of alkynes as reactants in organic synthesis, the synthesis of alkynes themselves has been much less developed. Figure 1 depicts the huge difference in the number and variety of protocols between alkyne preparation and reactivity, which generates a paradox that is difficult to solve. For the former, only acetylene and phenylacetylene are employed industrially in multiton amounts, and this is the reason why the vast majority of alkynes in commercial catalogues are propargyl alcohols, coming from the nucleophilic addition of acetylene and phenylacetylene derivatives to carbonyl groups (aldehydes and ketones).

The difficulties in obtaining a wide variety of alkynes are simply explained by the same reasons for their rich reactivity, i.e., the high energy of the C≡C bond, which obligates the use of highly energetic starting materials to achieve the desired alkyne product while circumventing undesired by-reactions of the reactive alkyne product. The main methods to obtain alkynes from other functional groups (not other alkynes) are arguably the double dehalogenation of vicinal halides with strong bases^5,6^ and the Seyferth-Gilbert homologation of aldehydes,^7^ and a clarifying probe of the limitations that current organic synthesis methodologies imposed to the synthesis of alkynes^3^ is that the third most used method for the synthesis of alkynes just combines the two reactions above, i.e., the Corey-Fuchs reaction (Figure 1B).^7^ These synthetic methods, including alternatives based on the use of aldehydes as starting materials,^7,8^ have in common that they use a strong base in stoichiometric amounts to generate the desired alkyne, since the latter is much less stable in acidic media.

Here, we propose the synthesis of terminal alkynes from alkenes. The alkene group is the most abundant hydrocarbon functional group in Nature and one of the cheapest to be produced industrially.^9,10^ Alkenes are, in general, very stable and compatible with many other functional groups, and they must lose only two H atoms to achieve the corresponding alkynes. Thus, it seems that the direct transformation of alkenes to alkynes would constitute a new avenue toward the synthesis (and later use) of alkynes. Indeed, Nature biosynthesizes alkynes from alkenes through dehydrogenase enzymes.^11^

The lack of reported studies on the direct transformation of alkenes to alkynes is somewhat puzzling, perhaps due to the difficulty of the transformation. We could only find three examples,^12−14^ backing 35–40 years ago, and the reproduction of these procedures in our hands (see next section) was very disappointing. The favorable thermodynamics of the dehydrogenation process (the energy associated with the C=C double bond in ethylene is 636 kJ·mol^–1^, the energy of the C≡C bond in acetylene is 837 kJ·mol^–1^, and the energy of the H–H bond is 435 kJ·mol^–1^; thus, the process is exothermic by 234 kJ·mol^–1^) does not operate by the lack of formation of H_2_, but H^+^ captured after a redox process. To circumvent this problem, we propose here a sequential, one-pot procedure, where one of the alkene H atoms is first replaced with a removable group (here silanes), to finally remove the second H atom by oxidation of the removable group (Figure 1C). This formal dehydrogenation reaction operates in one pot with commercially available reagents and under mild reaction conditions, with favorable thermodynamics. Despite we can advance that the scope is directed to terminal but not internal alkynes, and that yields are moderate to high, we hope that these results will stimulate the chemistry community to study the direct transformation of alkenes to alkynes.

## Results and Discussion

### Attempted Reproduction of Reported Precedents

**Figure 2:**
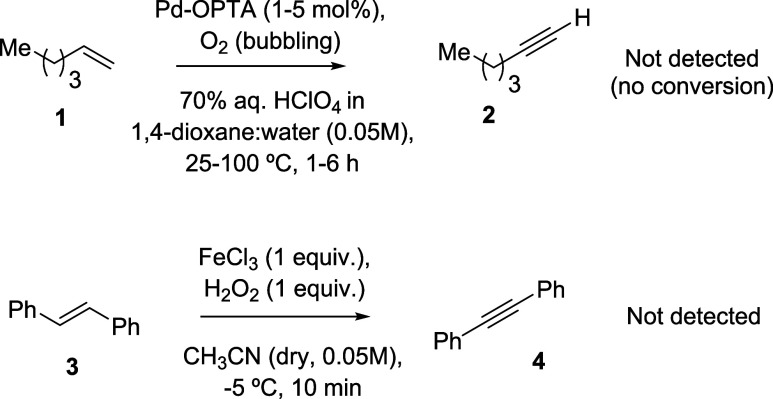
Reproduction attempts of reported precedents for the dehydrogenation of alkenes to alkynes.

The dehydrogenation of alkenes to alkynes was reported nearly 40 years ago with a Pd-supported *oligo*-*p*-phenyleneterephthalamide polymer (OPTA) catalyst in the presence of molecular oxygen O_2_ and perchloric acid HClO_4_ in ethanol- or dioxane-water solutions.^12^ Given the convenience of this procedure, we tried to reproduce the reported results. Since we were not able to find the exact preparation conditions for the polymer support, we employed the most similar one in the literature,^15^ and after supporting Pd under the reported conditions,^12^ we proceeded with the dehydrogenation reaction of 1-hexene **1** using this polymer-supported Pd catalyst. After several attempts, varying the reaction temperature, O_2_ feeding, and catalyst amount, we could not get any conversion of **1**, as shown in Figure 2, and alkyne **2** could not be even detected by gas chromatography (GC) analysis. A second related procedure where O_2_ was also used as a dehydrogenating agent was not reproduced here since it is not general but only applicable for particularly substituted anthracene and stilbene alkynes.^13^

We focused then on a third reported procedure where water peroxide H_2_O_2_ is used as a dehydrogenating agent.^14^ This procedure employs stoichiometric amounts of ferric chloride FeCl_3_ in combination with H_2_O_2_, in acetonitrile solutions, and the results in our hands under the reported reaction conditions (Figure 2, bottom)^14^ show that *trans*-stilbene **3** is not converted to diphenylacetylene **4**, but to different oxygenated products, which include stilbene epoxide and formaldehyde.

### Ru-catalyzed Dehydrogenative Silylation of Terminal Alkenes

At this point, we tested our envisioned strategy. The dehydrogenative functionalization of alkenes is a well-known process with many variants, including the dehydrogenative dismutation,^16^ borylations,^17^ thianthrenation,^18^ sulfonation,^19^ and thalliation^20^ reactions, and these reactions are efficient and often uncatalyzed, however, employ highly toxic reagents and difficult to oxidize atoms attached to the alkene. Thus, we chose here the dehydrogenative silylation reaction, which involves a much available, cheap, and less toxic silyl group, easily oxidable.^21^ This synthetic strategy follows our recent biomimetic^22^ approach with sulfonyl groups which, however, requires a combination of Ag and Fe catalysts, physically separated on the walls of a metal–organic framework (MOF), which imposes severe molecular dimension restrictions and high complexity to the catalytic design.^23^ Here, we employ commercially available Ru catalysts for the alkene dehydrogenative addition reaction, since Ru is well-known to catalyze dehydrogenation reactions through the formation of relatively stable Ru–H bonds, i.e., to promote hydrogen-borrowing mechanisms.^10,24^ We chose triethylsilane (HSiEt_3_) as the silane reactant since it has an intermediate volatility, which allows us to perform the reaction at different temperatures without silane losses and also allows its removal under vacuum. The results for the dehydrogenative silylation reaction of styrene **5** with HSiEt_3_ catalyzed by Ru compounds, employing 2-norbornene as a sacrificial alkene, are shown in Table 1.

**Table 1:**
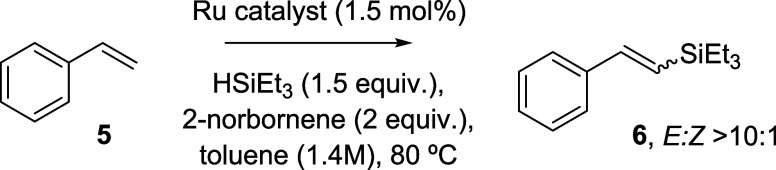
Catalytic Results for the Dehydrogenative Silylation Reaction of Styrene **5** to Give Vinyl Silane **6**^a^

entry	catalyst	time (h)	6 (yield, %)^b^
1	none	24	
2	RuCl_3_		
3	RuO_2_		
4	Ru(CO)H_2_(PPh_3_)_3_		14.8
5^c^	Ru(H)(Cl)(CO)(PPh_3_)_3_		66.4
6	Milstein’s catalyst		1.5
7	Gusev’s catalyst		14.1
8	RuCl_2_(C_14_H_12_NP)_2_		25.7
9^d^	Grubbs 1st gen		
10^d^	Grubbs 2nd gen		
11^d^	Hoveyda-Grubbs		
12	Ru_3_(CO)_12_		>99.0
13^e^		0.5	>99.0
**14**^e^^,^^f^			>**99.0 (98)**
15	Ru–C	24	
16	Ru–Al_2_O_3_		2.5
17^c^	Ru–Fe_2_O_3_		16.6
18^c^	Ru-SiO_2_		32.5
19^c^		4	22.7

a The *trans* to *cis* ratio for 6 is typically >10:1. GC results, product 6 was isolated from entry 12 in 98% yield. Each experiment was reproduced at least once. For the structure of the named catalysts, see Figure S1 (top).

b Between parentheses, isolated yield.

c A significant amount of the corresponding hydrosilylation product (alkyl silane) was found, typically 15–30%.

d The metathesis product plus polymerization products were found.

e 0.5 mol % of Ru_3_(CO)_12_.

f HSiEt_3_ (1.2 equiv) and 2-norbornene (1.6 equiv) were employed.

The dehydrogenative silylation reaction does not proceed in the absence of a catalyst, as expected (entry 1), and simple Ru salts such as RuCl_3_ and RuO_2_ do not catalyze the reaction (entries 2 and 3). However, different Ru complexes can catalyze the reaction. Ru(CO)H_2_(PPh_3_)_3_, with a preactivated Ru–H bond, gives the vinyl silane product **6** in 15% yield (entry 4), and the reaction boosts when Ru(H)(Cl)(CO)(PPh_3_)_3_, containing H, Cl, PPh_3_ and CO ligands, is employed as a catalyst (entry 5), with nearly total conversion and 66% selectivity to product **6**. However, the corresponding hydrosilylation product (alkyl silane, nondehydrogenative reaction) is also obtained (28.9%), which suggests that Ru–H complexes are not suitable to catalyze the reaction since the undesired hydrogenation reaction of the double bond is enhanced. Other Ru complexes containing either Ru–Cl and Ru-PPh_3_ bonds do not catalyze well the reaction (entries 5–11), including metathesis catalysts,^25^ because they can trigger the ring-opening metathesis reaction of the sacrificial alkene donor norbornene. In contrast, and gratifyingly, the simple Ru compound Ru_3_(CO)_12_ gave the desired product **6** in >99% yield (entry 12),^25^ and the amount of catalyst, silane and 2-norbornene, and also the reaction time, could be substantially diminished (entries 12–14, see Table S1 in the Supporting Information for a complete set of reaction conditions), to get product **6** in 98% isolated yield with just 0.5 mol % of Ru_3_(CO)_12_ in 30 min reaction time, in toluene solvent. Chlorinated solvents are also competent for the reaction but not other solvents such as THF or acetonitrile (Table S1).

Ru_3_(CO)_12_ is the cheapest Ru compound by Ru wt % in the market; thus, its high catalytic activity for the dehydrogenative silylation reaction is remarkable from an atom economy point of view. However, the coordinatively saturated structure of this Ru cluster, together with its tendency to decompose after CO removal, made us think that Ru_3_(CO)_12_ is just acting as a precatalyst and not the catalyst itself. To confirm this hypothesis, we performed kinetic experiments and, at the same time, we followed the dehydrogenative silylation reaction by in situ Fourier-transform infrared (FT-IR) spectroscopy and ultraviolet–visible (UV–vis) absorption spectrophotometry. To double-check the results, we used not only styrene **5** but also 1-heptene **7** as the starting alkenes. The results (Figures S1–S3) show that a clear induction time appears during the dehydrogenative silylation kinetic curve, as long as 30 min at 60 °C, and that this induction time decreases as the reaction temperature increases, to be ∼10 min at the optimized reaction temperature (80–100 °C, Figure S1, bottom), and give the desired vinyl silane products **6** and **8** in >99% yield after <1 h reaction time. The long induction time at lower reaction temperatures explains why the dehydrogenative silylation reaction requires 24 h to proceed at <60 °C, while higher temperatures dramatically decrease this reaction time. The in situ FT-IR measurements of the reaction at 80 °C (Figure S2) show the loss of the bridging CO ligands in the Ru_3_(CO)_12_ complex at ∼2000 cm^–1^ after 10 min of reaction time, just when the dehydrogenative silylation reaction starts, while the monocoordinated CO-Ru bonds remain. In accordance, the corresponding in situ UV–vis measurements (Figure S3, top) show the progressive displacement of the Ru_3_(CO)_12_ metal-to-metal transfer absorption band at 390 nm, associated with a Ru_3_ cluster,^26^ toward higher absorption energies, up to 355 nm, which indicates the loss of CO ligands. The calculation of the activation energy of the reaction for styrene **5** and 1-heptene **7**, of using an Arrhenius plot (Figure S4), gives a value of 14.2 and 14.6(1) kcal·mol^–1^, respectively, which informs that the reaction can proceed at moderate temperatures (i.e., 60 °C) once the catalytic active species are formed, after the induction time, and that both aromatic and alkyl alkenes engage equally well during the reaction. All of the results above strongly support that Ru_3_(CO)_12_ is not the active catalytic species but some other Ru complex formed in solution after the loss, at least, of the bridging CO ligands.

The possibility that a CO-free Ru species could be able to catalyze the dehydrogenative silylation reaction stimulated us to test Ru-supported species, since this would allow the recovery of the Ru species after the reaction. Unfortunately, the commercially available Ru–C and Ru–Al_2_O_3_ solids were merely inactive for the reaction (entries 15–16 in Table 1). These solids are constituted by Ru nanoparticles, which perhaps are too stabilized to engage in the reaction, due to the electron-rich hydrophobic surface of the support.^27^ For this reason, we then tested Ru nanoparticles supported on less electron-rich solids, prepared in our laboratory,^11^ in this case Ru–Fe_2_O_3_ and Ru-SiO_2_. Both solids were catalytically active for the reaction after 24 h of reaction time (entries 17–18). The fact that Ru-SiO_2_ is more active than Ru–Fe_2_O_3_ confirms that the catalytic activity of Fe can be considered negligible here. A decent yield of 32% to product **6** could be obtained with Ru-SiO_2_; however, significant amounts of the alkyl silane product were found (entry 18). These results, although unsatisfactory from a synthetic point of view, are promising in the search for a heterogeneous catalyst for the Ru-catalyzed dehydrogenative silylation reaction of alkenes, not reported yet to our knowledge. Besides, reflectance diffuse UV–vis analyses of the fresh and spent Ru-SiO_2_ catalyst (Figure S3, bottom) show the loss of the scattered plasmonic band associated with the supported Ru nanoparticles while preserving the bands at 230 and 290 nm, assignable to discrete Ru–O species,^28^ indicating the potential catalytic activity of oxidized Ru species during reaction, in reasonable agreement with the results obtained above with Ru_3_(CO)_12_ in solution.

After the in situ formation of the Ru catalytic species, the corresponding aromatic (product **6**) or alkyl vinyl silane (product **8**) is obtained in >99% yield in <1 h reaction time. These results are valid for designing the one-pot strategy toward the synthesis of the final terminal alkynes. Despite the Ru-catalyzed dehydrogenative silylation reaction did not work for internal alkenes, neither aryl nor alkyl (Figure S5, top), the implementation of other dehydrogenative reactions such as the dehydrogenative borylation of internal alkenes^29,30^ may lead to the synthesis of internal alkynes (see next section).

### Alternative Synthesis of the Vinyl Silane

We choose the dehydrogenative silylation of the alkene since it occurs under neutral reaction conditions, only H_2_ is liberated as a byproduct (and trapped by the sacrificial alkene), and Ru is a relatively inexpensive metal catalyst. However, other alternatives may be considered. We discarded the Heck silylation reaction since it requires silyl halides, which are much more unstable and expensive than silanes, produces the corresponding halide salt with a base, and employs typically much more expensive Pd as a metal catalyst (although Mn can be employed in some examples). Another alternative is the olefinic metathesis reaction with preformed internal vinyl silanes, and although this synthetic sequence (Figure S5, bottom) requires the use of an alkyne to prepare the vinyl silane compound **6′**, we expect to find, later on, another way to synthesize this internal vinyl silane if the route works. Compound **6′** was obtained in 75% GC yield with 0.005 mol % of a Pt catalyst;^31^ however, all our attempts to perform the metathesis reaction of **6′** with either 1-hexene **1** or styrene **5** unfortunately failed, after employing a variety of metathesis metal catalysts (Table S2, see Figure 6 for structures), some of them reported for the intramolecular metathesis reactions of trisubstituted vinyl silanes.^32,33^ The only products found were those corresponding to the self-coupling of the alkene (i.e., stilbene **SI-8**). Other vinyl silane was prepared in high purity (compound **SI-11**, Table S3), however, with the same bad results as for **6′** (Table S4).

At this point, a second alternative was considered, i.e., the dehydrogenative monoborylation reaction of alkenes.^30^ We reproduced the conditions of the dehydrogenative monoborylation reaction described for *cis*-stilbene **SI-8** and cyclododecene **SI-9**,^30^ and the catalytic results (Table S5) show that only the vinyl boronate of the latter could be obtained with this method, in good yield (93%). The oxidative dehydroelimination reaction of this vinyl was tested under the optimized reaction conditions described above for vinyl silanes (see ahead, i.e., Figure 4); however, a conversion of 44.9% and an overall yield of isolated product of just 4.0% was achieved. These data are interesting since they show that it is possible to obtain the alkyne from vinyl boronates, although in very low yields so far.

### Synthesis of Phenylacetylene 9 after Oxidative Dehydroelimination Reaction of Vinyl Silane 6

#### Optimization Results

The transformation of vinyl silane **6** to the corresponding alkyne, i.e., phenylacetylene **9**, employing as the starting material the optimized mixture in the step above (entry 14 in Table 1), without any treatment, was then studied. The results with different oxidants, Lewis acids, and base quenchers are shown in Table 2. The reactions were performed at room temperature (20 °C here) during 3 h of reaction time.

**Table 2:**
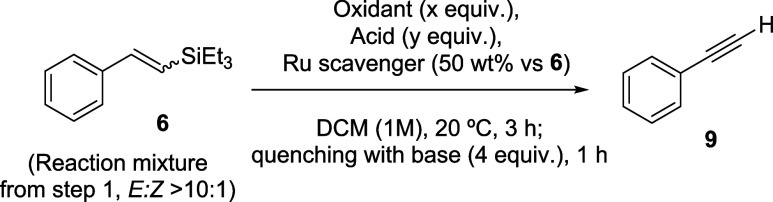
Results for the Oxidative Dehydrosilylation Reaction of the Reaction Mixture in the Previous Step (Entry 14 in Table 1), Containing Vinyl Silane **6**, to Give Phenylacetylene **9**^a^

entry	oxidant (equiv)	acid (equiv)	base (4 equiv)	Ru scavenger	Conv/sel 9 (%)^b^
1	PhIO (2.5)	SiCl_4_ (2.5)	NaOH (aqueous)		
2	PhIO (2.5)	AlCl_4_ (2.5)			
3^c^	PhIO (2.5)	BF_3_·OEt_2_ (2.5)			55.3/90.3
4	PhICl_2_				
5	PhI(OAc)_2_				20.6/-
6	^t^Bu-OOH (2.5)				66.9/-
7	KMnO_4_ (2.5)				14.6/-
8	PhIO (2.5)		NaHCO_3_ (aqueous)		48.6/90.6
9			Na_2_CO_3_ (aqueous)		32.1/80.6
10			KOAc (aqueous)		35.0/74.9
11	PhIO (1.2)	BF_3_·OEt_2_ (1.2)	NaOH (aqueous)		24.6/61.1
12	PhIO (1.5)	BF_3_·OEt_2_ (1.5)			48.4/48.4
13	PhIO (2.0)	BF_3_·OEt_2_ (2.0)			42.2/90.4
14	PhIO (2.5)	BF_3_·OEt_2_ (2.5)	(1 min)		31.2/75.3
15			(30 min)		51.8/71.3
16			(120 min)		52.2/90.2
17			(60 min)	charcoal (5 min)	>99.0/74.2
18				charcoal (60 min)	>99.0/79.8
**19**				**charcoal (120 min)**	>**99.0**/**82.1**
20^d^				SCX (60 min)	>99.0/80.4
21^e^				MPF-SiO_2_ (60 min)	>99.0/78.1

a GC results. Each experiment was reproduced at least once.

b GC results. Mass balance is completed with benzaldehyde, produced after oxidative breaking of product **9**.

c A similar result was obtained with in-house-made PhIO instead of the commercial reactant, and also when PhIO and BF_3_·OEt_2_ were combined previously, in a separated flask, dissolved in DCM, and stored for weeks in a fridge.

d SCX: Silica-bond tosic acid.

e MPF-SiO_2_: 3-Mercaptopropyl-functionalized silica gel.

The use of a high-valence iodinated compound as an oxidant was considered in the first place since the substitution of Si by iodonium in vinyl silanes is known.^34^ However, this Si-to-iodonium substitution is reported with the assistance of extremely reactive Lewis acid compounds such as O^+^(BF_4_)_3_,^34^ incompatible with the reaction mixture that we achieve after the dehydrogenative silylation reaction. Thus, we tested here the possibility of using a simpler combination of iodonium plus Lewis acid compounds to be quenched with an aqueous base and get the final alkyne. The combination of PhIO as an oxidant with SiCl_4_ or AlCl_4_ as an acid did not give any product (Table 2, entries 1 and 2); however, when BF_3_·OEt_2_ was used, a promising 55% conversion of **6** with 90% selectivity to phenylacetylene **9** was obtained after treatment of the oxidant mixture with aqueous NaOH for 1 h (entry 3).^35^ The mass balance was completed with benzaldehyde, coming from the oxidative breaking of phenylacetylene **9**. Remarkably, when PhIO and BF_3_·OEt_2_ were previously dissolved in a separate flask and stored for weeks in a refrigerator, a similar result was obtained. Thus, we can consider that the PhIO + BF_3_·OEt_2_ mixture is an oxidative dehydrosilylating agent, very easy to prepare, and storable (Figure S7). Other iodonium compounds such as PhIOCl_2_ and PhI(OAc)_2_ did not give any phenylacetylene **9** (entries 4–5), which indicates that the reaction is very specific for PhIO. to discard any impurity in the commercial PhIO reagent which could act here during the reaction and mask the results, we independently synthesized PhIO^36^ and tested the new sample in reaction, to give the same result. Other *O*-containing oxidants such as ^t^Bu-OOH and KMnO_4_ converted the vinyl silane **6**, although not alkyne **9** (entries 6–7). At this point, we tested different quenching bases dissolved in water, including NaHCO_3_, Na_2_CO_3,_ and KOAc, and all of them gave moderate conversions and good selectivity to **9** (entries 8–10). A decrease in the amount of PhIO and BF_3_·OEt_2_ translates into a decrease of the final yield of **9** (entries 11–13), as also does a different NaOH quenching time (entries 14–16).

The maximum yield of **9** obtained so far was 45% (entry 3), and any of the reaction variations could increase this value. Since the Ru(CO)_*x*_ catalyst is still present in the reaction mixture, we thought that perhaps the Ru complex could hamper the oxidative elimination reaction; thus, we decided to eliminate the Ru from the solution before the addition of the reagents, by employing a metal scavenger. Rewardingly, when commercially available active carbon (charcoal) was added to the mixture and stirred for just 5 min before the addition of the oxidant mixture, both the conversion and selectivity to **9** increased dramatically, to 99% and 74%, respectively (entry 17). The better selectivity to **9** found for the oxidative elimination after removal of some Ru confirms that some active Ru traces may catalyze the decomposition of vinyl silane **6** under oxidative conditions. Longer stirring times slightly increased the conversion value, to finally achieve a yield of **9** > 80% (entries 18–19). Other commercially available metal scavengers such as silica-bond tosic acid (SCX) and 3-mercaptopropyl-functionalized silica gel were equally effective as charcoal (entries 20–21).

Inductively coupled plasma-optical emission spectrometry (ICP-OES) of the scavenging charcoal, after filtration, shows that only 8% of Ru is removed from the solution. However, kinetic experiments (Figure S8) show that this 8% constitutes more than half of the catalytically active Ru for the dehydrogenative silylation reaction. The trapping of Ru occurs independently of the moment of addition of the charcoal, either before or after the induction time, since the induction time occurs regardless charcoal is present or not, and the initial reaction rate is very similar. These results indicate that charcoal does not trap the starting Ru_3_(CO)_12_ but the active Ru(CO)_*x*_ species, perhaps cationic, which makes sense considering the better trapping of positively charged metal species on negatively charged surfaces such as charcoal (or thiol-containing surfaces) and the results obtained above with Ru-supported species.

### One-Pot Synthesis of Terminal Alkynes from Alkenes

**Figure 3:**
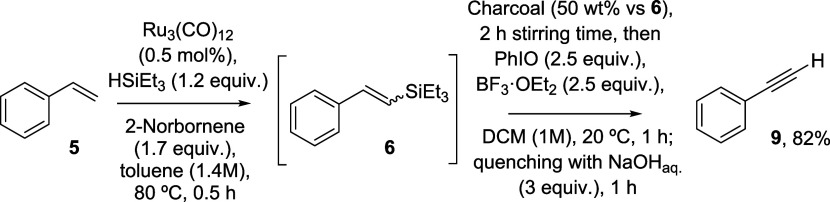
One-pot synthesis of phenylacetylene **9** from styrene **5**, in 4.5 h of reaction time, employing exclusively commercially available catalysts and reagents.

The results above built the necessary one-pot reaction conditions to achieve phenylacetylene **9** directly from styrene **5**. Thus, the one-pot reaction was attempted, and a gratifying 82% yield of **9** was obtained from **5** after an accumulated 4.5 h reaction time during the two steps, as shown in Figure 3.

**Figure 4:**
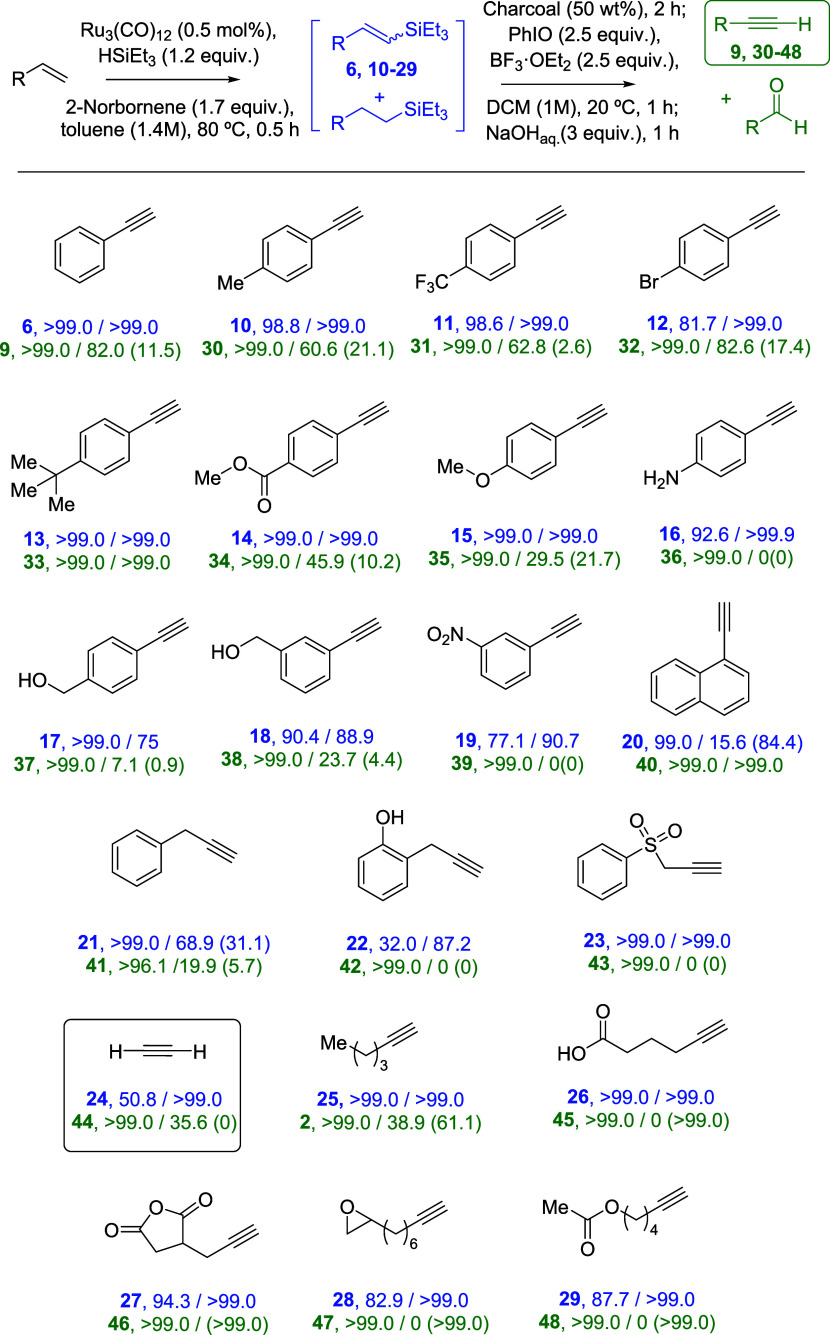
Scope of alkynes prepared by the one-pot dehydrogenative silylation-oxidative dehydroelimination reaction under optimized reaction conditions. Results in blue: conversion/selectivity of the dehydrogenative step (%); results in green: conversion/selectivity of the elimination step (%); selectivity to the main byproducts between parentheses. Each experiment was reproduced at least once. The loss of alkyne selectivity is mainly due to polymerization reactions.

Figure 4 shows the synthesis of other terminal alkynes from the corresponding alkenes. The results show the yields obtained for the vinyl silane intermediates **10**–**29** (in green) and the final alkynes **30**–**49** (in blue). The vinyl silanes were independently isolated to confirm their formation (see the Supporting Information for characterization details), while the formation of the alkynes was confirmed after comparison with commercial samples by mass spectrometry (GC-MS) and ^1^H, ^13^C and distortionless enhancement by polarization transfer (DEPT) nuclear magnetic resonance (NMR). The amount of main byproducts is also indicated in Figure 4, which corresponds to the hydrosilylated products during the first step (see above) and to aldehydes coming from the oxidative breaking of the alkyne product during the second step. Note that these aldehydes are indeed products from the alkyne, which can be considered a further extension of the one-pot transformation from the alkene.

A variety of *para*-substituted styrene derivatives react in high yields to the corresponding phenylacetylene derivatives, having methyl (product **30**, 60% yield), trifluoromethyl (product **31**, 62% yield), bromide (product **32**, 82% yield), *tert*-butyl (product **33**, 99% yield), methyl ester (product **33**, 46% yield) and methoxy (product **34**, 30% yield) substitutents. The latter gave significant amounts (10–20%) of the corresponding benzaldehyde byproducts. We tried to purify the terminal alkyne products **30**–**33**; however, the product was lost when removing the solvents (volatiles) in all cases, which was expected for these low-boiling-point products. Nevertheless, mass spectrometry characterization shows the typical fragmentation for these very well-known compounds, and we corroborated the structure by comparison with commercially available samples. The aniline derivative (product **36**) gave high yields of the silane intermediate (product **16**, 92% yield) but polymerized during the second step, under the oxidative reaction conditions. The same polymerization reaction is observed for the silane intermediates **17**–**19** (obtained in 70–75% yield), giving low amounts of the final alkynes **37**–**39** (<20% yield). A naphthalene substituent in the vinyl silane bears well the oxidative conditions (product **40**, quantitative yield from the vinyl silane **20**) but the hydrosilylation reaction occurs to a high extent in the first step (84% yield). Note that our methodology overrides in economic terms other processes for the synthesis of alkynes such as the Sonogashira coupling (which starts already from an alkyne, to give another alkyne), since an alkene typically costs, at least, 1 order of magnitude less than the corresponding alkyne (for instance, 100 mL of phenylacetylene costs ≈100 euros, while 1 L of styrene costs ≈50 euros in commercial catalogues and for the same purity).

Alkyl alkenes are also reactive during the one-pot procedure. The benzylic vinyl silanes **21**–**22** and the related vinyl silane **23** were obtained with good selectivity (>68%), but the corresponding alkynes **41**–**43** were obtained in low yield or not observed, due to polymerization reactions. In contrast, low-grade ethylene containing 1 wt % of acetylene as an impurity, thus simulating raw ethylene after cracking processes, gave acetylene **44** in 17% yield (without considering the starting acetylene impurity) and with 67% selectivity. In this case, the reaction was performed in an autoclave to avoid any loss of volatile reactants and products. Note that the intermediate vinyl silane **24** is obtained with >50% yield and total selectivity under the general reaction conditions since, in this particular reaction, the alkene (ethylene) and the Ru-catalyzed silylation system reside in different phases (gas and liquid, respectively) which, although slows the reaction rate, also avoids undesired by-reactions. This result is remarkable since we could not find any other procedure in the literature to directly transform ethylene to acetylene **44** beyond typical petrochemistry conditions. The synthesis of acetylene **44** from ethylene is convenient not only for economic but also for purity and safety reasons, at least at the laboratory scale, since a cylinder of acetylene gas is only sold by specialized commercial houses in a mixture with acetone (to avoid flammability) and in relatively high amounts (i.e., 10 L) while, in contrast, ethylene is sold by most of the chemical providers in the market in neat form and, if desired, in small amounts (i.e., 1 L).

1-Hexene **1**, the alkene reported 40 years ago to be transformed into 1-hexyne **2** with a Pd-supported OPTA polymer catalyst (see Figure 1, top),^12^ was also tested under our one-pot reaction conditions, to give a 39% yield. The intermediate vinyl silane **25** was obtained in quantitative yield, and the loss of yield is due to the formation of valeraldehyde (1-pentanal) in 61% yield during the second step of the one-pot reaction. Independent reactions with either 4-methoxystyrene or alkyne **14** under the oxidative reaction conditions confirm the exclusive formation of aldehyde from the alkyne and not from the alkene (Figure S9). The rest of the alkyl alkenes tested gave exclusively the aldehyde products resulting in the oxidative breaking of alkynes **45**–**48**, in >80% yields after the two steps. These results, despite not giving the alkynes as final products, show that not only the synthesis of the intermediate vinyl silanes **26**–**29** but also the transformation to alkyne works in high yields to give the corresponding aldehydes as final products.

### Reaction Mechanism of the Oxidative Dehydrosilylation Reaction

The uniqueness of PhIO to perform the oxidative dehydroelimination reaction prompted us to study the reaction mechanism. For that, in situ ^1^H, ^11^B, ^13^C, and ^19^F NMR studies of the reaction were performed after the sequential addition of all reactants.

**Figure 5:**
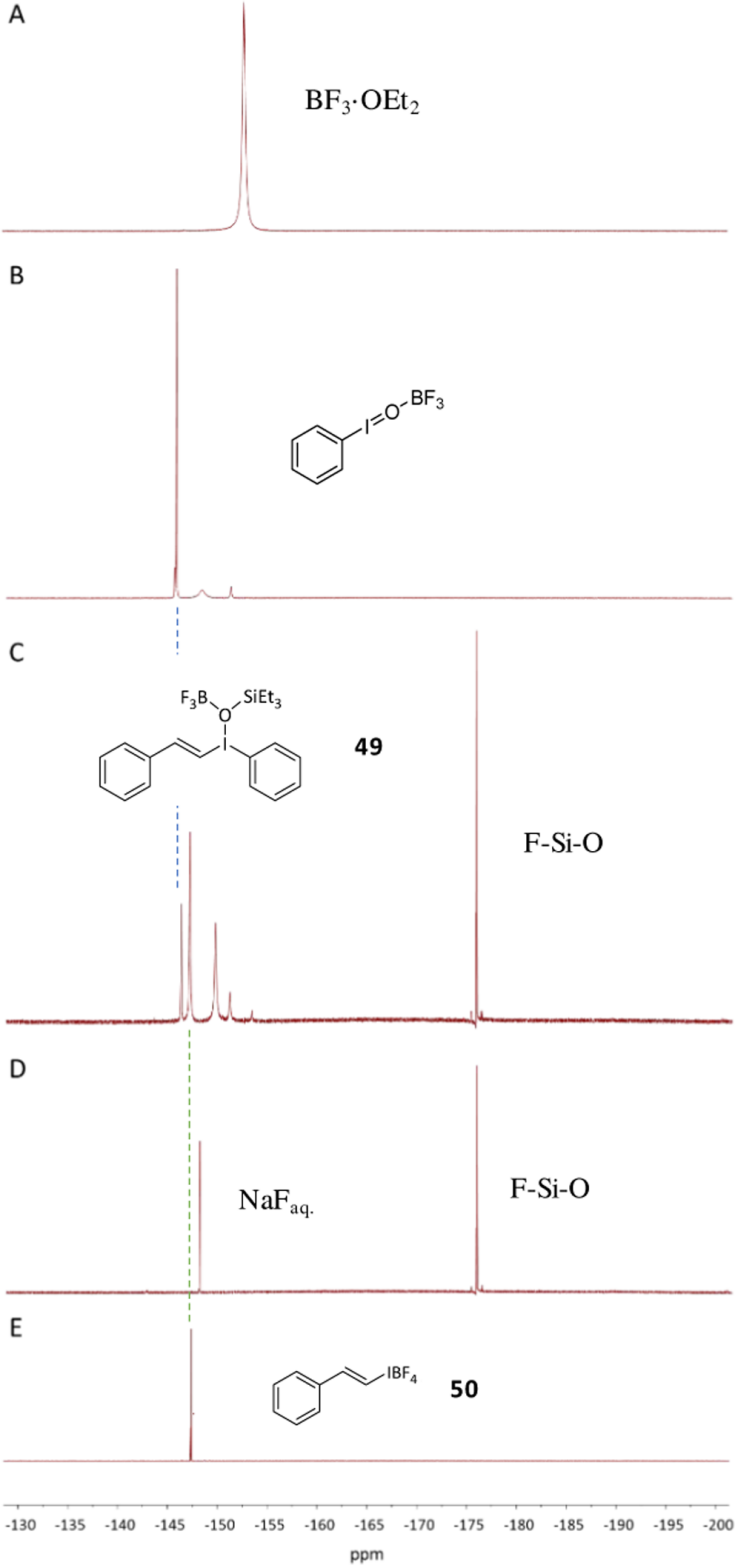
^19^F NMR spectra of (A) BF_3_·OEt_2_, (B) + PhIO (1 equiv), (C) + vinyl silane **6** (to form **49**), (D) + NaOH, and (E) independently prepared compound **50**.

The first step is the equimolar addition of BF_3_·OEt_2_ to PhIO. The quantitative ^1^H NMR spectrum shows that BF_3_·OEt_2_ loses the OEt_2_ ligand rapidly (the ether signals shift to free OEt_2_, compared with the starting BF_3_·OEt_2_ spectrum, Figure S10), to give a spectrum compatible with the new species F_3_B·OIPh, formed in 66% yield according to this NMR measurement. Particularly informative are the displacements of the ^11^B (Figure S11) and ^19^F signals, shown in Figure 5, which support the proposed transformation of BF_3_·OEt_2_ to F_3_B·OIPh in 66% yield (see Figure S11).

**Figure 6:**
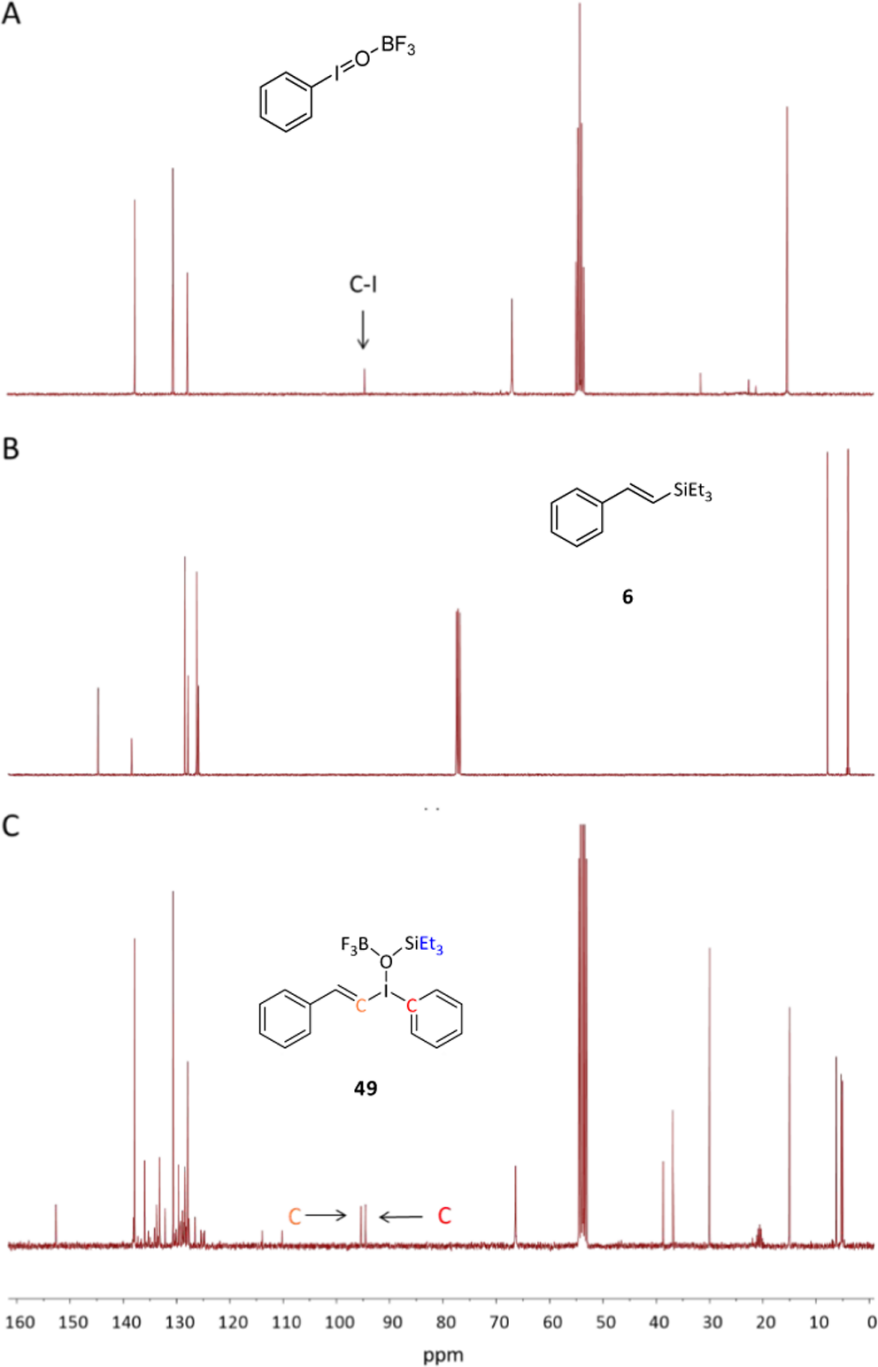
(A) ^13^C NMR spectra of the equimolar mixture of BF_3_·OEt_2_ and PhIO; (B) chromatographically purified vinyl silane **6**; and (C) the corresponding mixture of both (to form **49**), indicating the diagnostic signals for the different species.

Then, chromatographically purified vinyl silane **6** was added. The ^11^B and ^19^F NMR signals slightly changed, to give spectra compatible with a similar species to F_3_B·OIPh (Figures S11 and 5). However, the ^1^H and ^13^C NMR spectra of the new mixture were completely different. The ^1^H NMR shows a dramatic change in the alkene signals of **6** after the addition of the F_3_B·OIPh reagent (Figure S10), and the corresponding ^13^C NMR in Figure 6 shows that one of the vinyl carbon atoms downshifts from a value of ∼125 to ∼95 ppm, close to the aromatic C–I bond of PhIO. The corresponding DEPT NMR spectrum with a pulse at 135° (^135^DEPT) unambiguously confirmed that this new signal at ∼95 ppm corresponds to a quaternary new C bond, plausibly the new vinyl CH-I bond (Figure S12).

The reaction was also followed by ^29^Si NMR, and the corresponding spectra (Figure S13) show that HSiEt_3_ (spectrum A)^37^ evolves to vinyl silane **6** with a slight downshield of the signal (just +3 ppm, spectrum B), which further downshields (+35 ppm, spectrum C) after generating the proposed intermediate **49**, in accordance with the high inductive effect of the iodonium cation. Finally, the addition of NaOH produces the formation of Et_3_SiOH (19 ppm),^38^ Et_3_SiOSiEt_3_ (9 ppm),^39,40^ and diverse siloxy and fluorosiloxy compounds.^41^

With all of the NMR data in hand, one can propose that the intermediate species formed after the addition of the PhIO + BF_3_·OEt_2_ mixture to **6** is the corresponding vinyl iodonium species **49**, whose proposed structure is shown in Figures 5 and 6 (see also Figures S10–S12). To confirm this hypothesis, we independently prepared the very similar compound **50**,^42^ and not only the corresponding ^1^H, ^11^B, ^13^C, ^19^F, and ^29^Si NMR spectra (Figures 5, 6, and S10–S14) but also the DEPT spectrum (Figure S12) nicely fit the spectra of the proposed intermediate **49**. When the isolable iodonium compound **50** was submitted to the eliminating reaction conditions with aqueous NaOH, phenylacetylene **9** was formed in >95% yield (see ahead), which strongly supports the intermediacy of the proposed compound **49** during the reaction.

**Figure 7:**
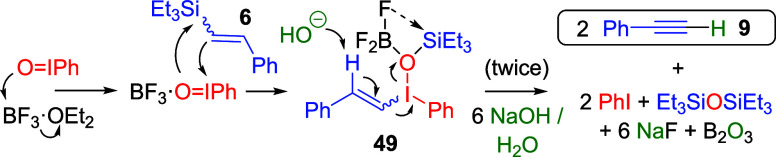
Proposed mechanism for the synthesis of intermediate **49** and phenylacetylene **9**.

Figure 7 shows the proposed mechanism for the oxidative dehydrosilylation reaction of viny silane **6** with the stable mixture of BF_3_·OEt_2_ + PhIO. In situ monitoring of the reaction by UV–vis experiments (Figure S15) shows that the well-differentiated BF_3_·OEt_2_ bands at 370 and 320 nm disappear after the addition of PhIO, which indicates the formation of F_3_B·OIPh. Besides, the addition of vinyl silane **6** to F_3_B·OIPh generates a new band at 290 nm with a shoulder at 310 nm, which nicely fits the main band of the independently prepared vinyl iodonium **50**. When NaOH_aq_. is added, the band disappears. These results strongly support the proposed intermediate **49** during the oxidative dehydrosilylation reaction of **5** to **9**.^43^

Further reactive experiments were carried out to confirm the proposed mechanistic sequence. The addition of aqueous NaOH to **50** quantitatively produces phenylacetylene **9** (Figure S16, top), which further supports the formation of **49** during the process. The elimination step could proceed after the attack of the base to the acidic α-vinyl H atom,^44^ with the assistance of the exceptionally good leaving iodonium group,^45^ which then reduces to PhI. However, NaOH pellets do not trigger this process and water must be present in the reaction medium (Figure S16, bottom).^46^ The aqueous basic solvent not only provides the chemical environment to generate and dissolve the oxide species of both B (B_2_O_3_) and Si (siloxane species), as well as NaF (all of these species are compatible with the observed ^11^B, ^19^F, and ^29^Si spectra after aqueous treatment, see Figures 5 and S11), with the associated favorable enthalpy to the overall processes, but also provides, by exchange, the final H atom of phenylacetylene **9**. When the isotopically labeled intermediate **49-*****d***^***2***^ was prepared from styrene-*d*^*3*^ (**5-*****d***^***3***^) and treated with NaOH_aq._, following our optimized reaction conditions, the obtained product was mainly **9**, not the deuterated alkyne. In contrast, when **49** (formed in situ) or **50** (independently prepared and isolated) were treated with NaOH dissolved in D_2_O, 67% of **9-*****d***^***1***^ was found (Figure S16, bottom; see Figure S17 for the GC-MS spectrum). These results strongly support the exchange of the H atom of phenylacetylene **9** in the biphasic aqueous reaction medium.^47^

## Conclusions

The one-pot synthesis of terminal alkynes, including acetylene, from alkenes proceeds with a Ru-catalyzed dehydrogenative silylation reaction followed by an oxidative desilylation reaction enabled by in situ-formed F_3_B·OIPh, after a final basic aqueous treatment. The addition of charcoal to trap the catalytically active Ru species, after the first step, increases the yield and selectivity of the alkyne. Other methods to avoid the trapping of Ru have been considered. The intermediate F_3_B·OIPh reagent is easily prepared by simply mixing PhIO and BF_3_·OEt_2_ in DCM, and stored in a refrigerator for months. The mechanistic study of the reaction shows that the Ru catalyst for the dehydrogenative addition is formed in situ during the reaction, that it can be supported on solids, and that the resulting vinyl silane is fully dehydrogenated to the acetylide during the oxidative reaction. The overall process uses commercially available reagents, proceeds under practical mild conditions, and can be carried out in less than one working day.

## Experimental Section

### Preparation of F_3_B·OIPh

The complex was prepared by adding PhIO (2.5 mmol) and DCM (1 mL, 2.5 M) in a 2 mL glass vial equipped with a magnetic stirrer and leaving shaking for 5–15 min until complete dissolution. Then, BF_3_·OEt_2_ (2.5 mmol, 1 equiv) is added very slowly under magnetic stirring at room temperature until completely dissolved. A yellow solution is obtained (see Figure S7). ^1^H NMR (300 MHz, CD_2_Cl_2_) δ: 8.14 (d, *J* = 7.6 Hz, 2H), 7.71 (t, 1H), 7.56 (d, *J* = 7.7 Hz, 2H), 3.49 (q, *J* = 7.0 Hz, 3H), 1.17 (t, *J* = 7.0 Hz, 6H). ^13^C NMR (75 MHz, CD_2_Cl_2_) δ: 138.0 (CH), 130.9 (CH), 128.1 (CH), 94.7 (C).

### Ru-Catalyzed Dehydrogenative Silylation Reaction

The vinyl silane products were prepared following the reaction described in Figure 4. The Ru_3_(CO)_12_ catalyst (0.0007 mmol, 0.5 mol %) and 2-norbornene (2.3 mmol, 1.6 equiv) were introduced in a 2 mL glass vial equipped with a magnetic stirrer, and toluene (1 mL, 1.4 M) was added. The mixture was purged with N_2_, and the corresponding alkyne (1.4 mmol, 1 equiv) and HSiEt_3_ (1.7 mmol, 1.2 equiv) were added. The vial was capped, and the mixture was placed in a preheated oil bath at 80 °C, and magnetically stirred for 30 min. The resulting mixture was characterized by GC (employing *n*-dodecane as an external standard), GC-MS, and NMR after removing the volatiles under vacuum (see the Supporting Information).

### Oxidative Dehydrosilylation Reaction

The synthesis of phenylacetylene **9** from isolated vinyl silane **6** was performed after adding complex F_3_B·OIPh dissolved in DCM (1.7 mmol, 2.5 equiv., previously prepared from PhIO and BF_3_·OEt_2_ as indicated above) to a solution of **6** (0.7 mmol) in DCM (0.7 mL, 1M, from the first step) in a 2 mL vial equipped with a magnetic stirrer. The mixture was magnetically stirred for 1–24 h, and then, the resulting mixture was quenched by the addition of NaOH (4 equiv, 2.8 mmol) in water (1 mL, 2.8M) for 1 h. The mixture was extracted with DCM and dried over Na_2_SO_4_. The resulting mixture was analyzed by GC (employing *n*-dodecane as an external standard) and GC-MS, and also compared with commercial samples when available.

### One-Pot Dehydrogenative Silylation-Oxidative Dehydrosilylation Reaction of Alkenes to Alkynes

Ru_3_(CO)_12_ catalyst (0.0007 mmol, 0.5 mol %) and 2-norbornene (2.3 mmol, 1.6 equiv) were introduced in a 10 mL round-bottom flask equipped with a magnetic stirrer, and toluene (1 mL, 1.4M) was added. The mixture was purged with N_2_, and the corresponding alkyne (1.4 mmol, 1 equiv) and HSiEt_3_ (1.7 mmol, 1.2 equiv) were added. The mixture was placed in a preheated oil batch at 80 °C and magnetically stirred for 30 min. This mixture was then cooled and stirred with active charcoal (50 wt % respect to the vinyl silane, typically 80 mg) for 2 h and then filtrated to remove the solids. Then, complex F_3_B·OIPh dissolved in DCM (3.5 mmol, 2.5 equiv., previously prepared from PhIO and BF_3_·OEt_2_ as indicated above) was added and the resulting mixture was magnetically stirred at room temperature for 1 h. After that time, the resulting mixture was quenched by the addition of NaOH (4.2 mmol, 3 equiv) in water (1.5 mL, 2.8M) for 1 h. The mixture was extracted with DCM and dried over Na_2_SO_4_. The resulting compounds were analyzed by GC (employing *n*-dodecane as an external standard) and GC-MS, and also compared with commercial samples when available.

### One-Pot Dehydrogenative Silylation-Oxidative Dehydrosilylation Reaction of Ethylene to Acetylene **44**

The general procedure above was followed but used in this case a steel autoclave reactor equipped with a magnetic stirrer, a capillary inlet/outlet sampling, and a manometer. After the necessary reagents for the dehydrogenative silylation reaction were added, the autoclave was flushed with low-grade ethylene to remove any air, and the pressure was set at 5 bar of ethylene. The reactor was placed in a preheated oil bath at 80 °C and magnetically stirred for 30 min. Then, both the gas and liquid phases of the mixture were analyzed by GC-MS, finding that the vinyl silane intermediate is exclusively in the liquid phase (also checked by ^1^H NMR). Then, the necessary reagents for the oxidative dehydrosilylation reaction were added, and the resulting mixture was magnetically stirred for 1 h and quenched by the addition of NaOH for another 1 h, keeping the autoclave closed. DCM was added, and both the gas and organic liquid phases of the mixture were analyzed by GC-MS. Calibration with ethylene-acetylene samples was carried out to quantify the results.

## Supporting Information Available

Experimental generalities and details, product characterization, and NMR copies (Figures S1–S15) (Table S1) (PDF)
