# Supplementary material for: One-Pot Synthesis of Terminal Alkynes from Alkenes
Source: JACS Au. 2024 Aug 5;4(8):3284–94. doi: 10.1021/jacsau.4c00599 (PMC11350724; doi:10.1021/jacsau.4c00599)

Supporting Information (SI) for the manuscript:

One-pot synthesis of terminal alkynes from alkenes

Cristina Bilanin,[◊,#] Amravati S. Singh,[◊,#] Lluis Martínez-Belenguer[◊] and Antonio Leyva-Pérez.[◊,*]

[◊] Instituto de Tecnología Química (UPV-CSIC), Universitat Politècnica de València-Agencia Estatal Consejo Superior de Investigaciones Científicas, Avda. de los Naranjos s/n, 46022 Valencia, Spain.

[#] These authors contributed equally.

Table of Contents

Experimental Section	SI-3
Figures S1-S17	SI-4
Table S1-S5	SI-21
Characterization of isolated compounds	SI-26
NMR copies	SI-32

Experimental Section

General.

Reagents were obtained from commercial sources and used without further purification unless otherwise indicated. Anhydrous solvents were obtained from a resin-exchanger apparatus. Reactions were performed in conventional round-bottomed flasks or sealed vials equipped with a magnetic stirrer. All products were characterized by gas chromatography-mass spectrometry (GC-MS), proton (^{1}H), carbon (^{13}C) and distortionless enhancement by polarization transfer (DEPT) nuclear magnetic resonance (NMR) spectroscopy. Gas chromatographic analyses were performed in a Shimadzu GC-2025 instrument equipped with a 25 m capillary column of 1% phenylmethylsilicone using *n*-dodecane as an external standard. GC/MS analyses were performed on a spectrometer (Agilent GC 6890 N coupled with Agilent MS-5973) equipped with the same column as the GC and operated under the same conditions. ^{1}H, ^{13}C, ^{11}B, ^{19}F NMR and DEPT were recorded in a 300 MHz (or 400 MHz when available) instrument using $CDCl_3$, CD_3CN or toluene-D_8 containing TMS as internal standard as a solvent, unless otherwise indicated. Fourier transform infrared (FT-IR) spectra of the compounds were recorded on a spectrophotometer equipped with an attenuated total reflection instrument (JASCO FT/IR-4700), measuring from 400 to 4000 cm^{-1} by impregnating the windows with a dichloromethane solution of the compound and leaving to evaporate for 1 min on the ATR crystal before analysis. If the product is solid, the analyte is directly measured by compacting the solid on the spectrophotometer window before analysis. UV-Vis absorption spectrophotometry measurements were recorded on a Cary 300 UV-Vis spectrophotometer (UV0811M209, Varian) under air at room temperature, in a quartz cell of 1.0 cm optical path length. Inductively coupled plasma-atomic emission spectroscopy (ICP-AES) of the metal content of the samples was determined after disaggregating the solids in aqua regia and later diluted before analysis. Diffuse reflectance UV-Visible spectrophotometry (DR UV-vis) were recorded on a spectrophotometer equipped with an integrating sphere in the region of measurement comprised between 190 and 1100 nm, at room temperature. The solid was placed in a quartz cell with 1 mm path length; the layer can therefore be regarded as infinitely thick, as required by the Kubelka-Munk theory, and absorbance values (Al) were calculated from reflectance ones (Rl) according to the Kubelka-Munk transformation: Al = (1-Rl) 2/2Rl.

Figures

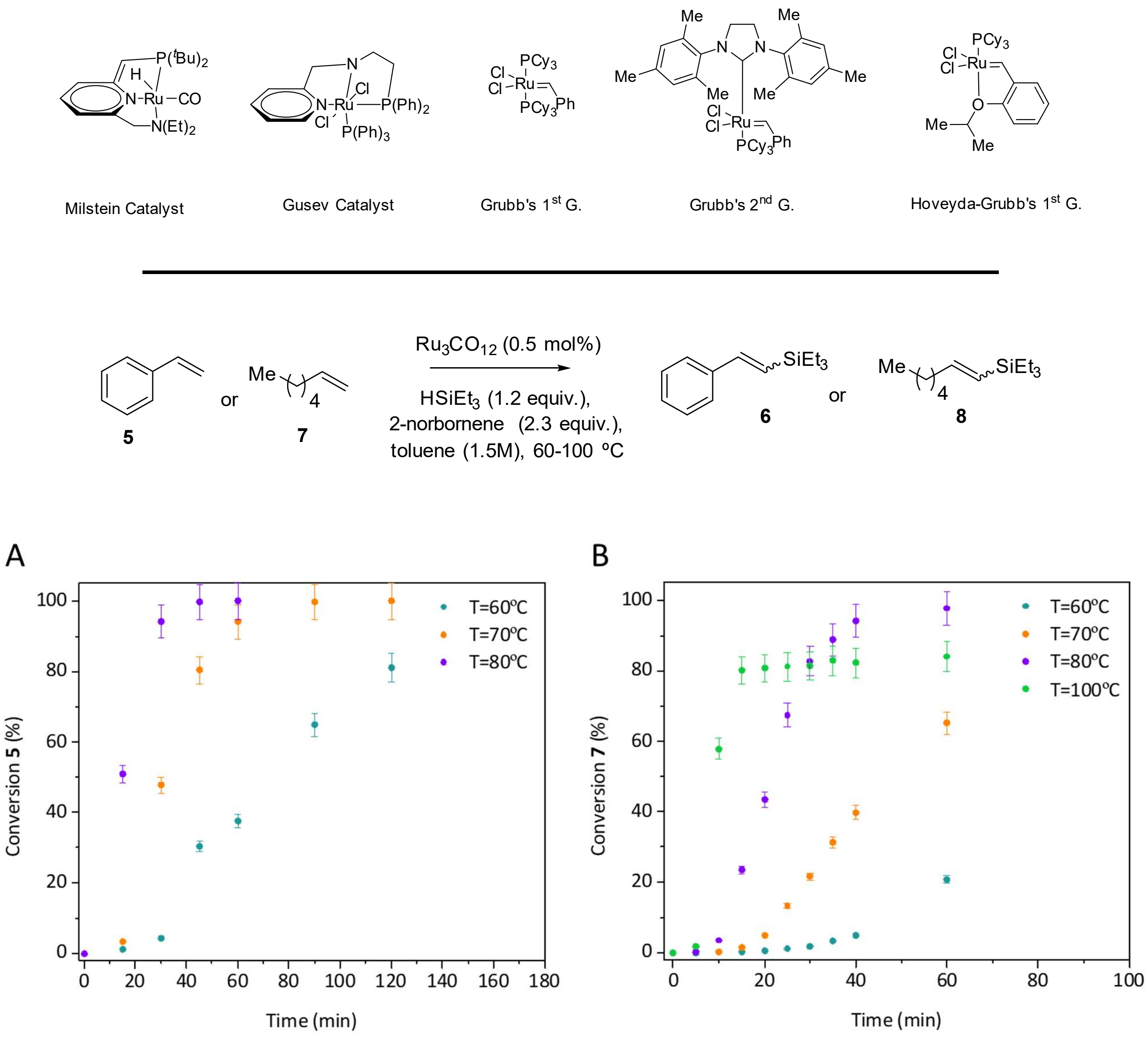

Figure S1. Top: Structure of the named ruthenium complexes used as catalysts for the dehydrogenative silylation reaction. Bottom: Kinetic plots at different reaction temperatures for the dehydrogenative silylation reaction of A) styrene **5** or B) 1-heptene **7** with $HSiEt_3$, catalyzed by $Ru_3(CO)_{12}$ (0.5 mol%) and with 2-norbornene as a sacrificial alkene, under the indicated reaction conditions. The *trans* to *cis* ratio for products **6** and **8** is typically >10:1. GC results. Error bars account for a 5% uncertainty.

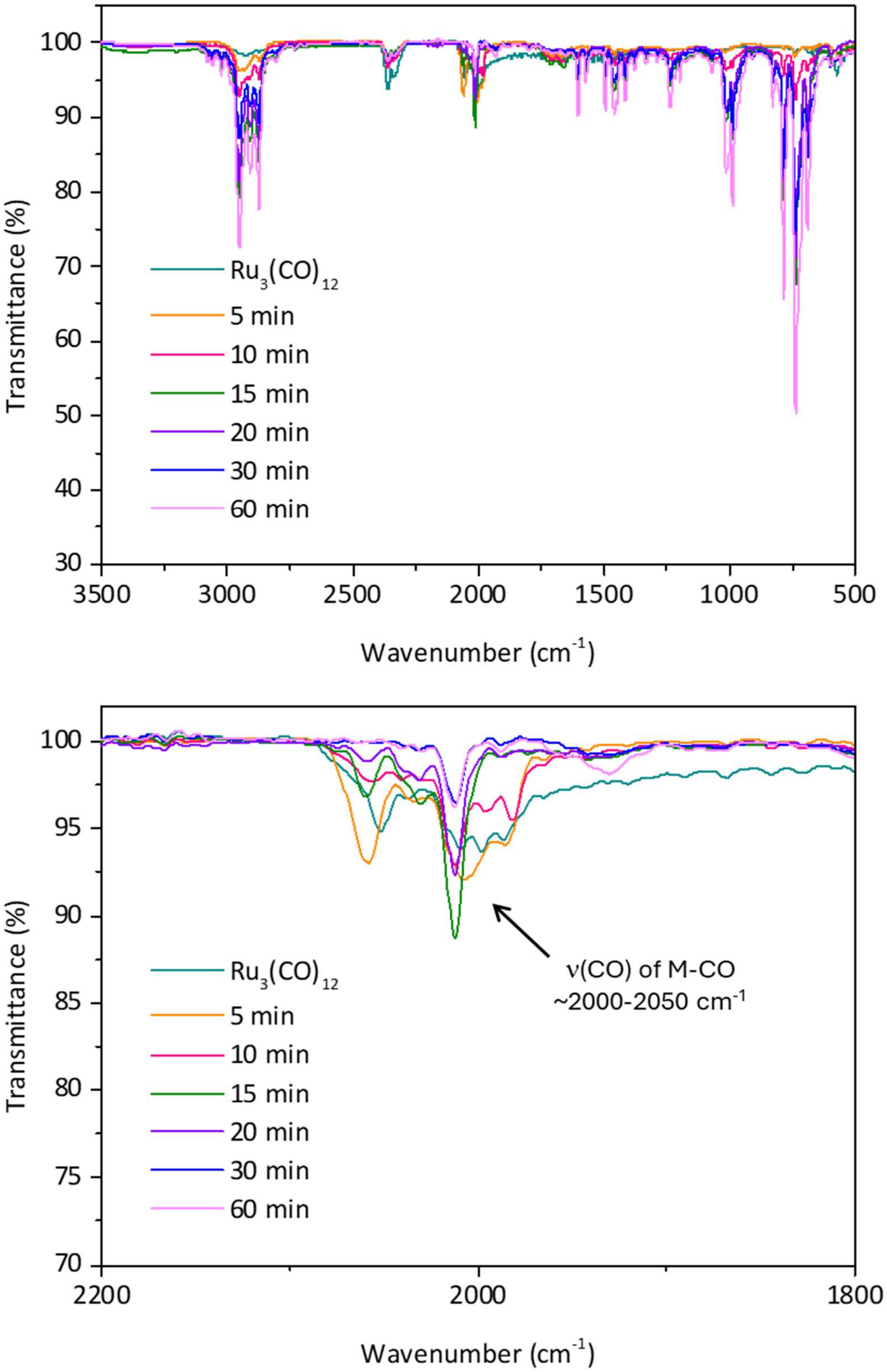

Figure S2. In-situ Fourier-transform infrared (FT-IR) spectra during the dehydrogenative silylation reaction of styrene **5** with $HSiEt_3$. The graph below magnifies the diagnostic area.

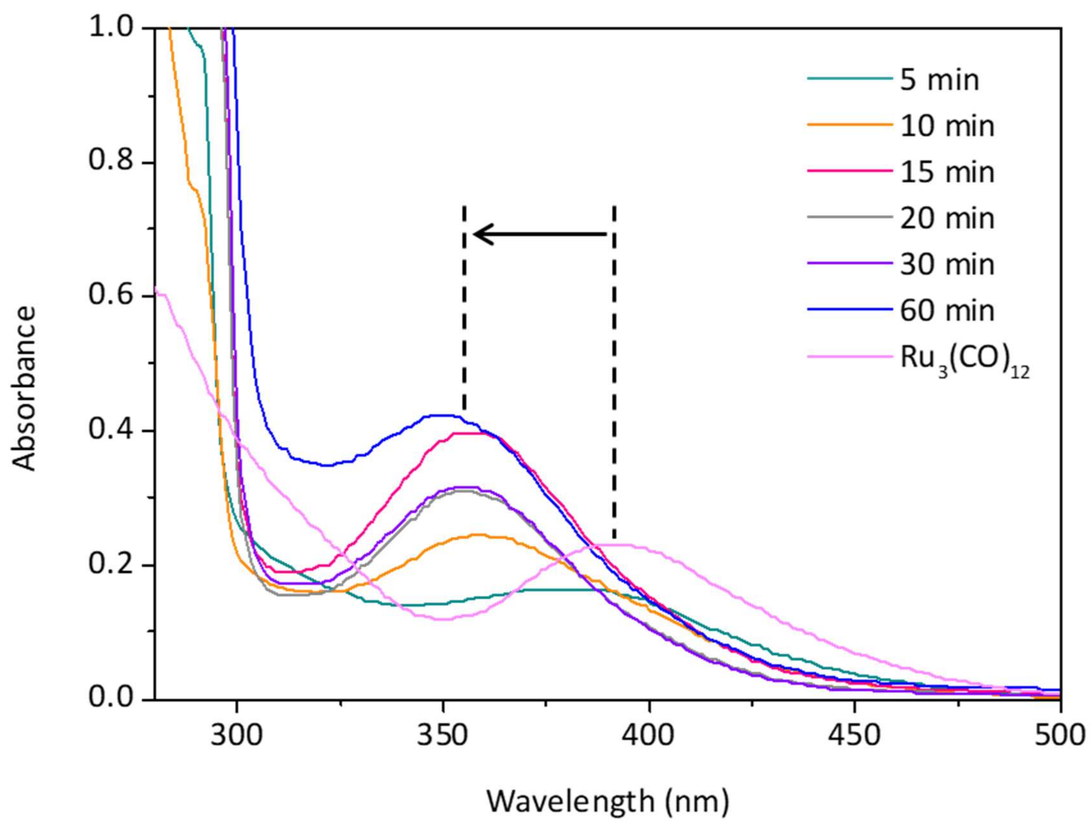

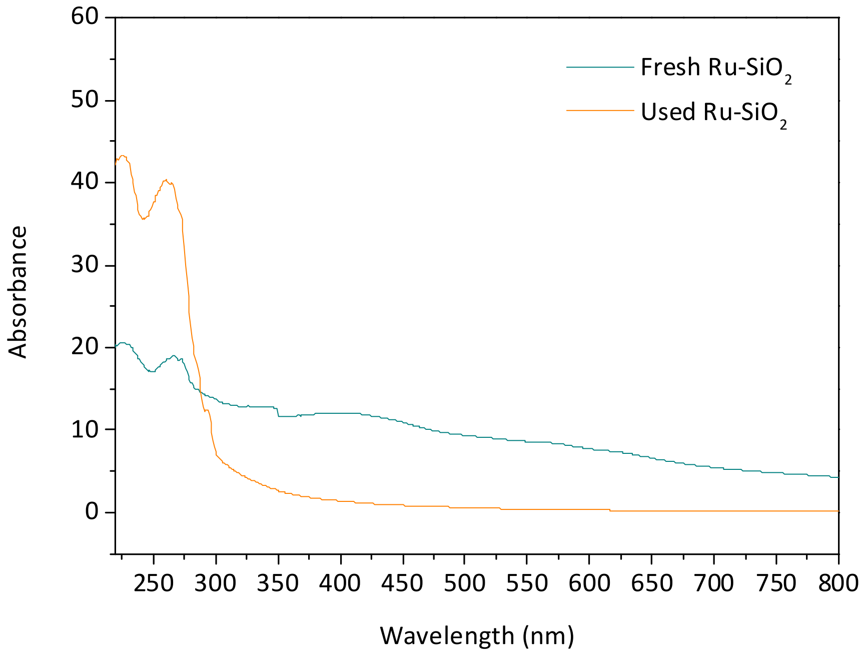

Figure S3. Top: In-situ ultraviolet visible (UV-vis) spectra during the dehydrogenative silylation reaction of styrene **5** with $HSiEt_3$. The disappearance of the starting $Ru_3(CO)_{12}$ band during the induction time (10 min) is clearly observed. Bottom: Reflectance diffuse UV-vis spectra of the fresh and spent Ru-SiO_2 during the dehydrogenative silylation reaction of styrene **5** with $HSiEt_3$. The peak at 350 nm is an artifact due to the lamp change.

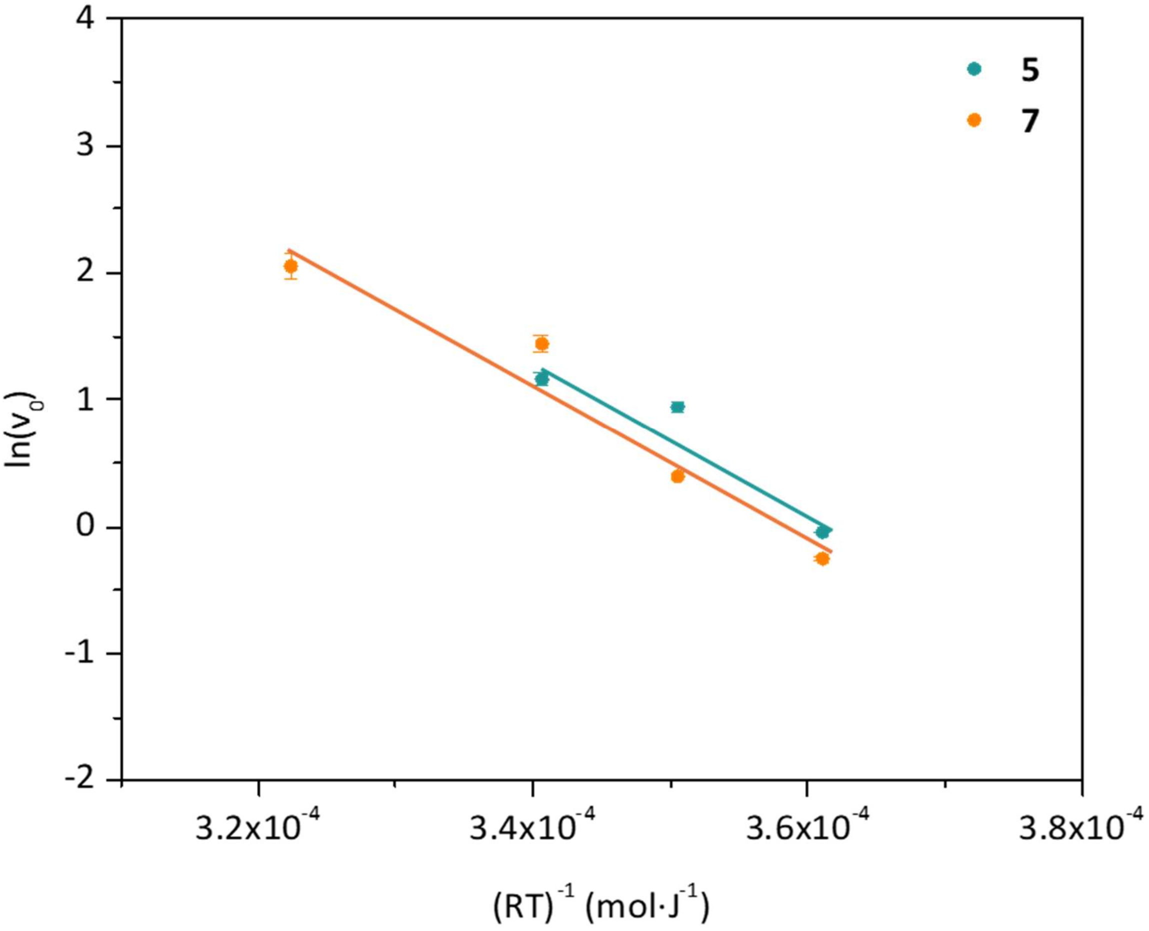

Figure S4. Calculation of the activation energy for the dehydrogenative silylation reaction of styrene **5** and 1-heptene **7** with $HSiEt_3$, catalyzed by $Ru_3(CO)_{12}$ (0.5 mol%) and with 2-norbornene as a sacrificial alkene. Error bars account for a 5% uncertainty. Regression values for styrene **5**: $\ln(v_0)=-59374(RT)^{-1}+21.509$, $R^2=0.89$; regression values for 1-heptene **7**: $\ln(v_0)=-61245(RT)^{-1}+21.956$, $R^2=0.95$.

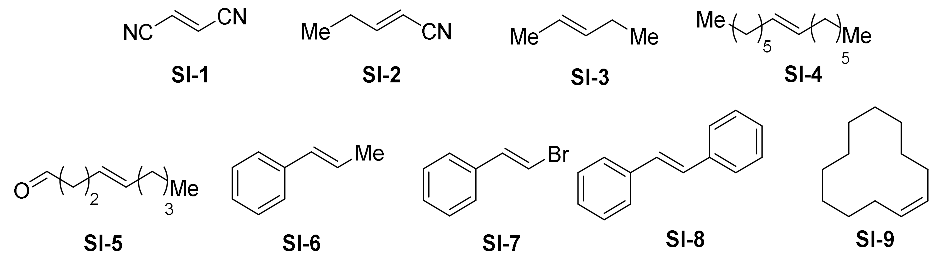

1. Prepare α-vinilsilane.

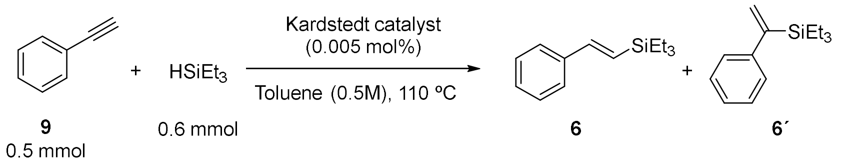

2. Potential olefin metathesis reaction.

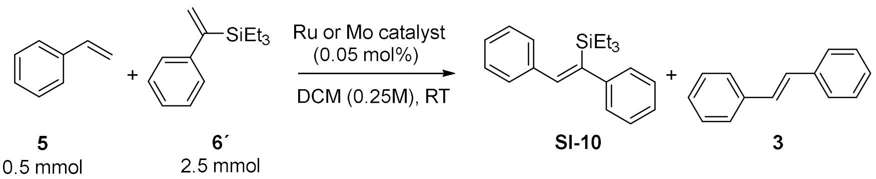

3. Potential alkyne formation.

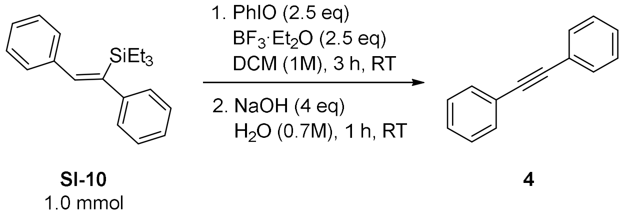

Figure S5. Top: Failed attempts for the Ru-catalyzed dehydrogenative silylation reaction of different internal alkenes. Bottom: Outline of the possible formation of internal alkyne **4**.

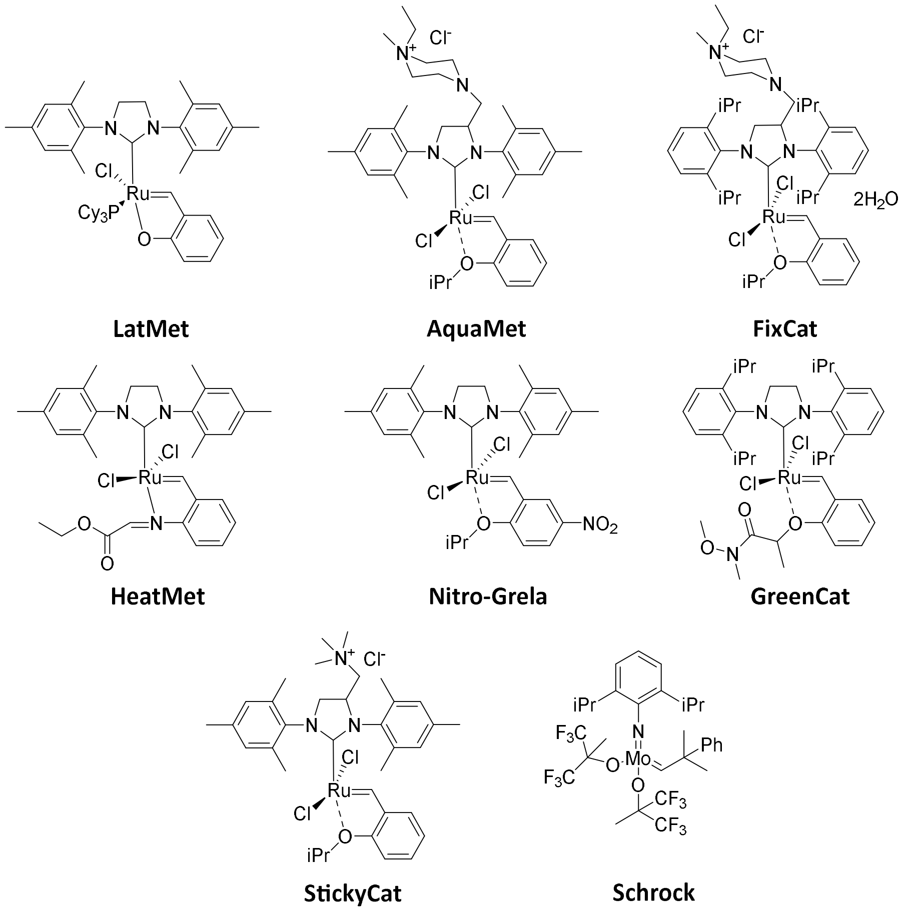

Figure S6. Structure of the ruthenium and molybdenum complexes used as catalysts for the metathesis reaction.

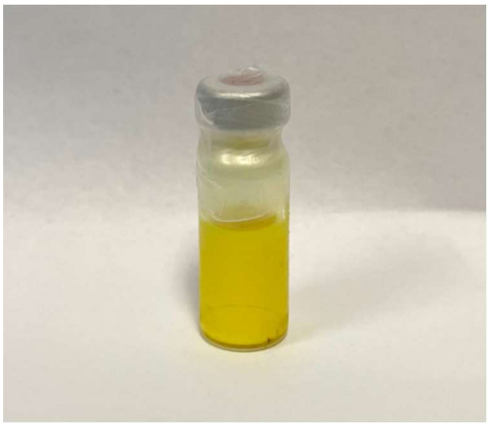

Figure S7. Photograph of a 2-mL vial with the PhIO + $BF_3 \cdot OEt_2$ mixture in dichloromethane solution (1M), which preserves complete reactivity for the formation of alkynes after storage in a fridge for 19 weeks.

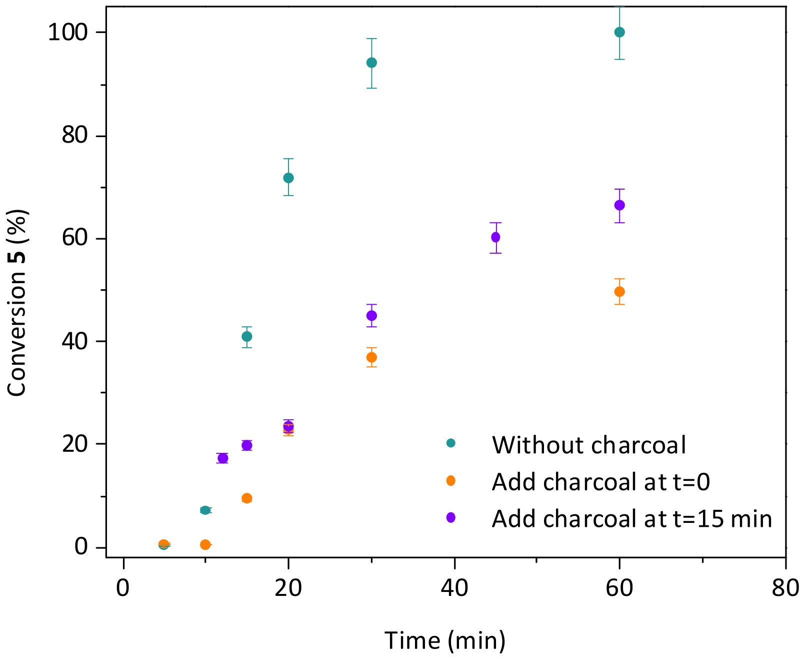

Figure S8. Kinetic plots for the dehydrogenative silylation reaction of styrene **5** with $HSiEt_3$, catalyzed by $Ru_3(CO)_{12}$ (0.8 mol%) and with 2-norbornene as a sacrificial alkene, in the presence or not of charcoal (50 wt% vs **5**). The *trans* to *cis* ratio for **6** is typically >10:1. GC results. Error bars account for a 5% uncertainty.

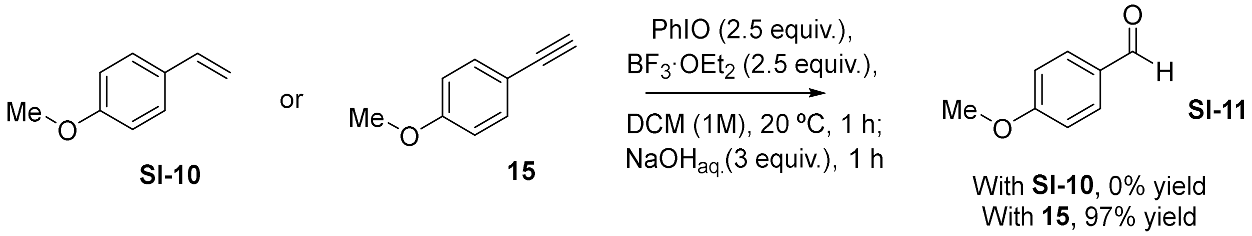

Figure S9. Reactive experiments for the oxidative breaking of either 4-methoxy styrene **SI-10** or 1-ethynyl-4-methoxybenzene **15** under the oxidative reaction conditions of step 2. Charcoal is not added since Ru is not present. GC yields. Alkene **SI-10** was recovered after the reaction.

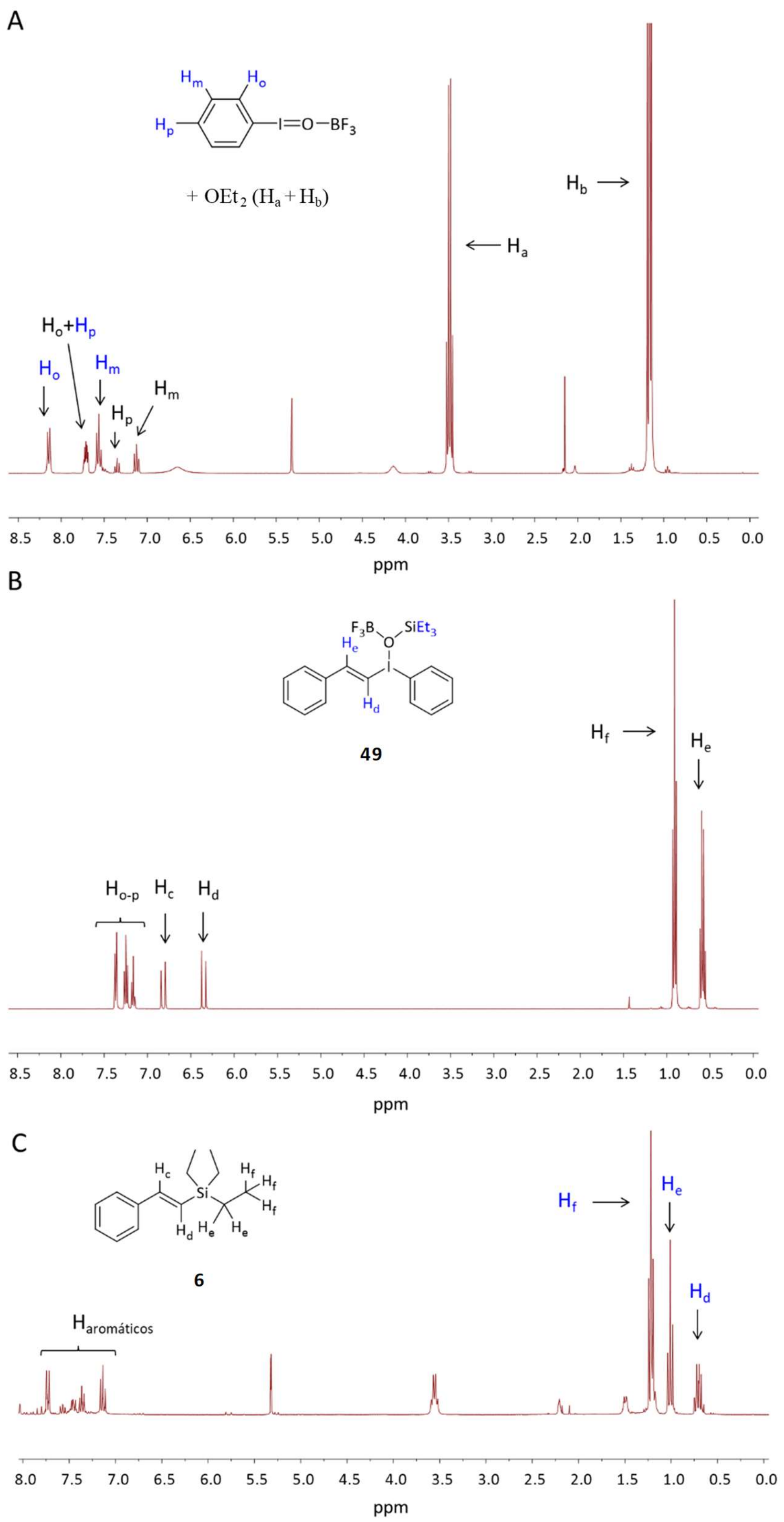

Figure S10. ^{1}H NMR spectra of A) $BF_3{\cdot}OEt_2$ + PhIO (1 equiv.), B) + vinyl silane **6** to give the proposed intermediate **49**, and C) + NaOH, to give product **6**. The diagnostic signals for the different species are indicated.

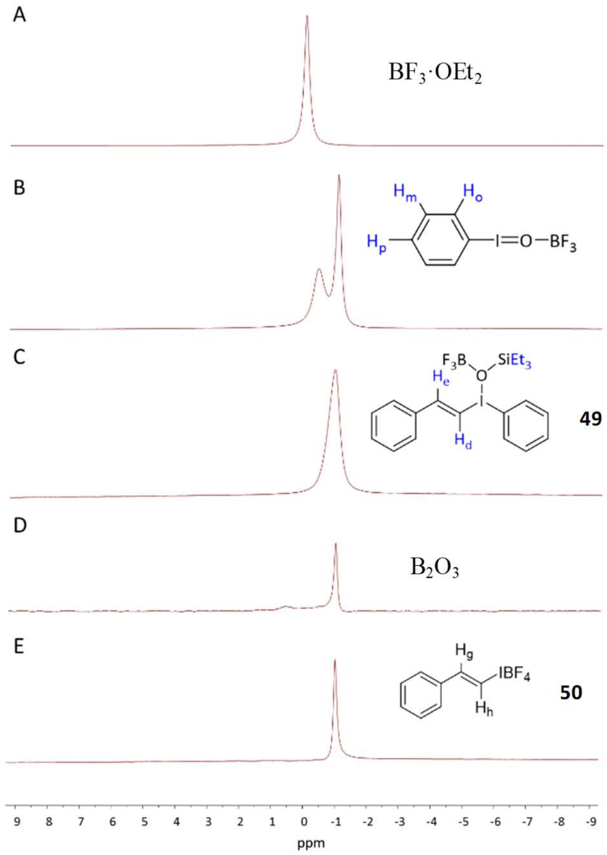

Figure S11. ^{11}B NMR spectra of A) $BF_3·OEt_2$, B) + PhIO (1 equiv.), C) + vinyl silane **6**, D) + NaOH, and E) independently prepared and isolated vinyl iodonium **50**.

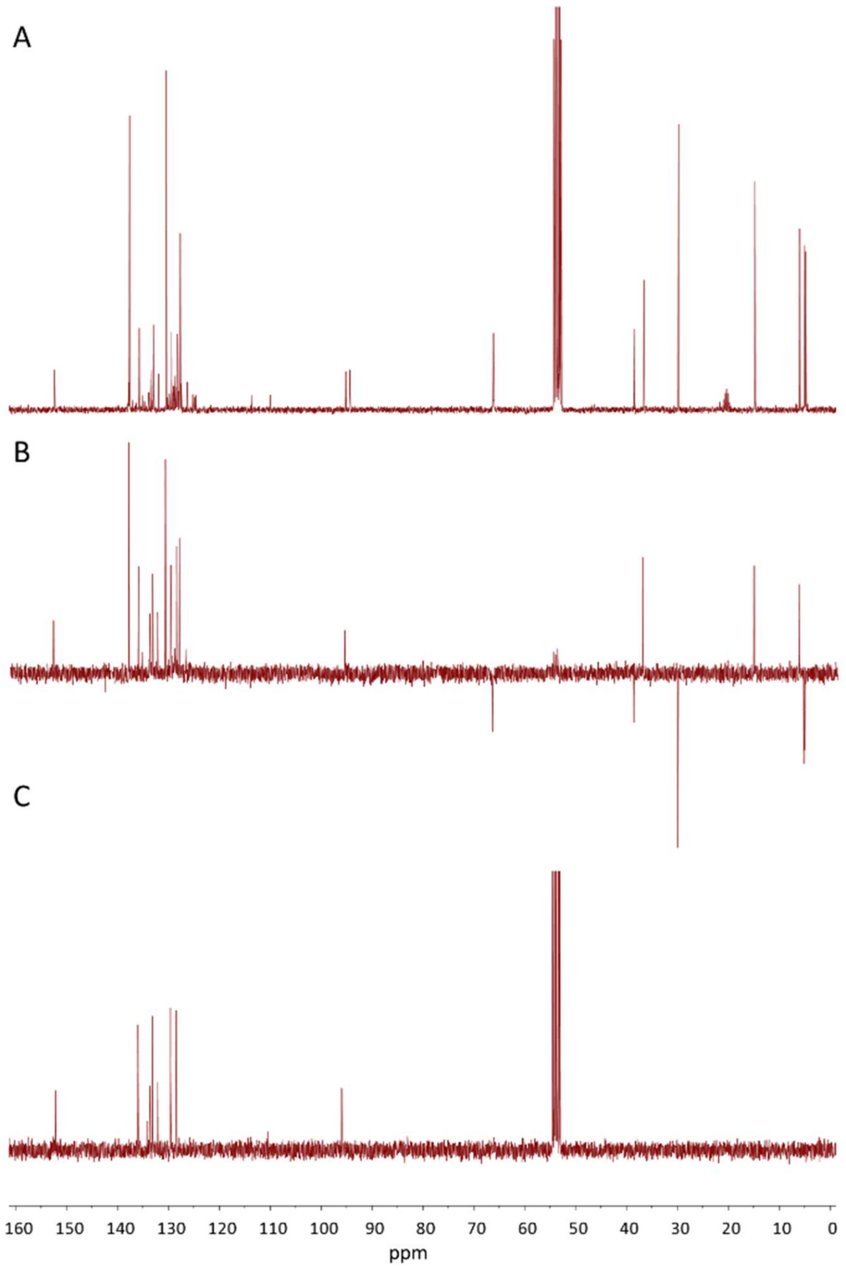

Figure S12. A) ^{13}C and B) ^{135}DEPT NMR spectra of the $BF_3 \cdot OEt_2$ + PhIO + vinyl silane **6** mixture, compared to C) the ^{13}C NMR of the independently prepared and isolated vinyl iodonium **50**.

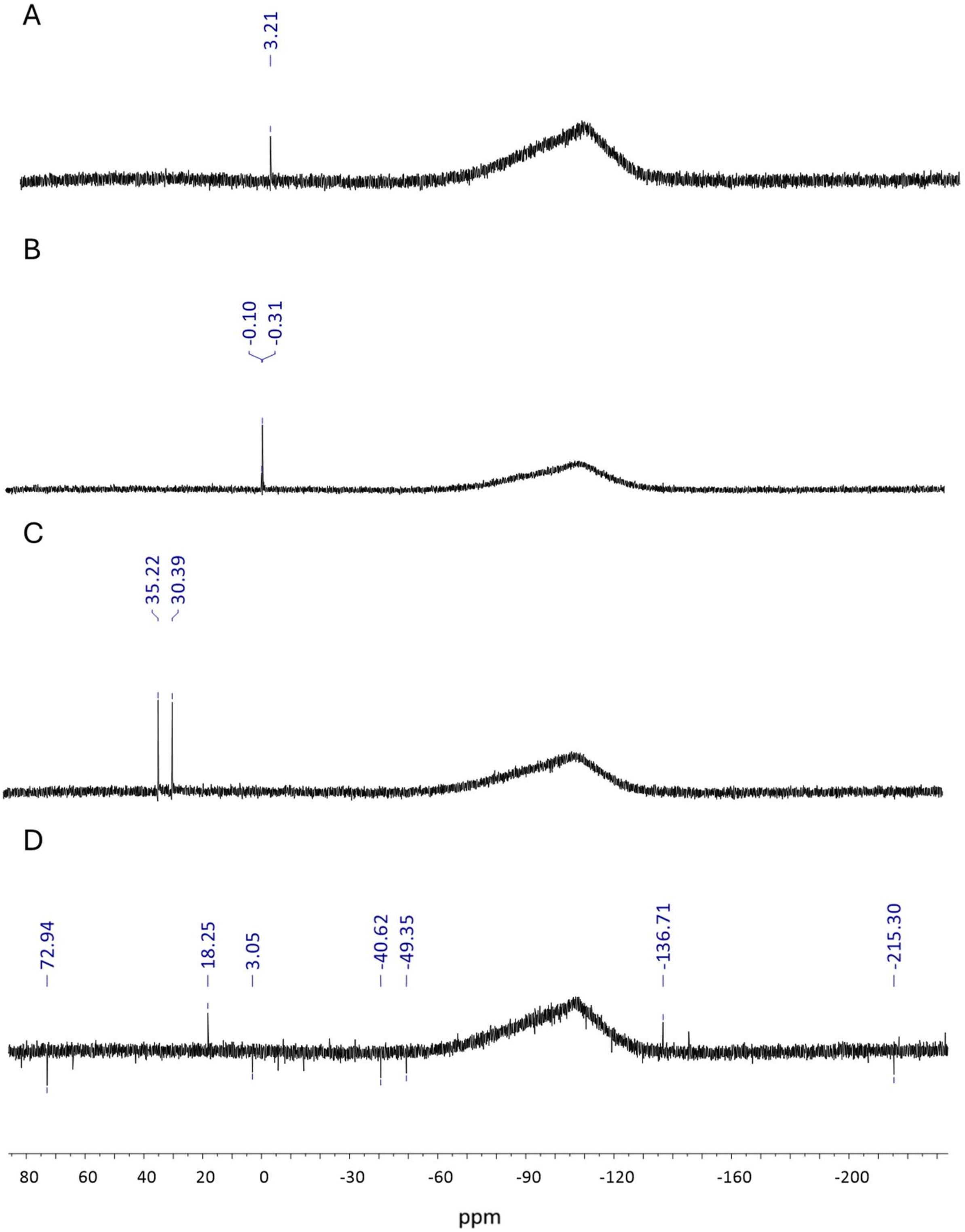

Figure S13. ^{29}Si NMR spectra of A) $HSiEt_3$, B) vinyl silane **6** (to form **49**), C) vinyl silane **6** + PhIO·BF_3, and D) + NaOH.

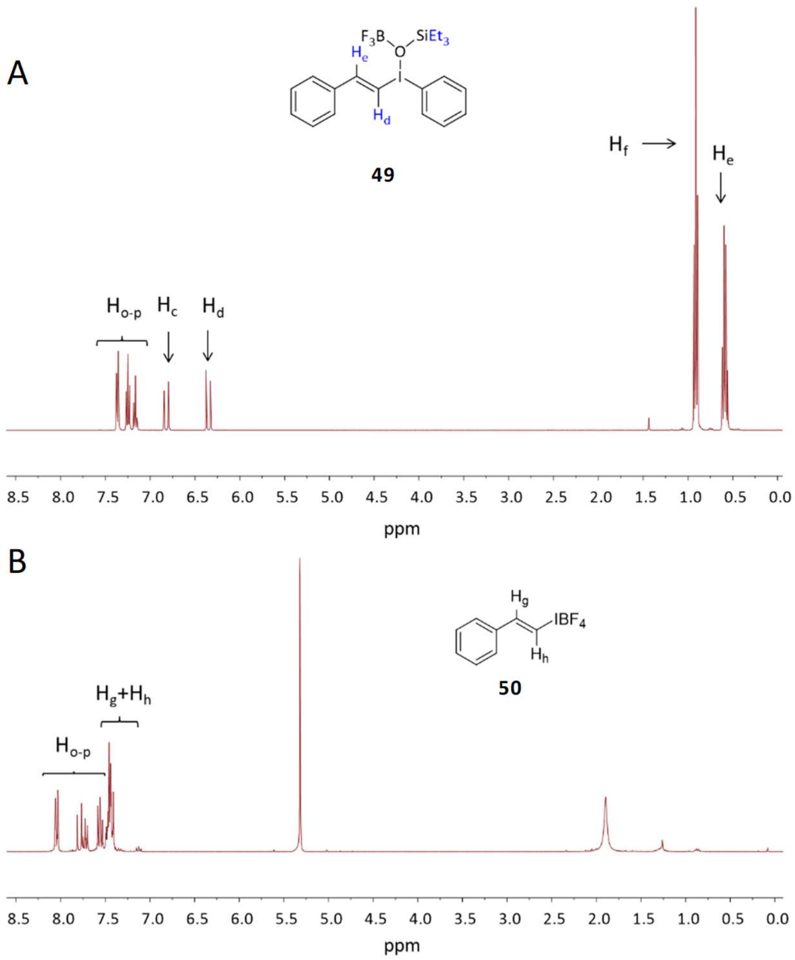

Figure S14. ^{1}H NMR spectra of A) the proposed intermediate **49** compared to B) the independently synthesized and isolated iodonium compound **50**, indicating the diagnostic signals for the different species.

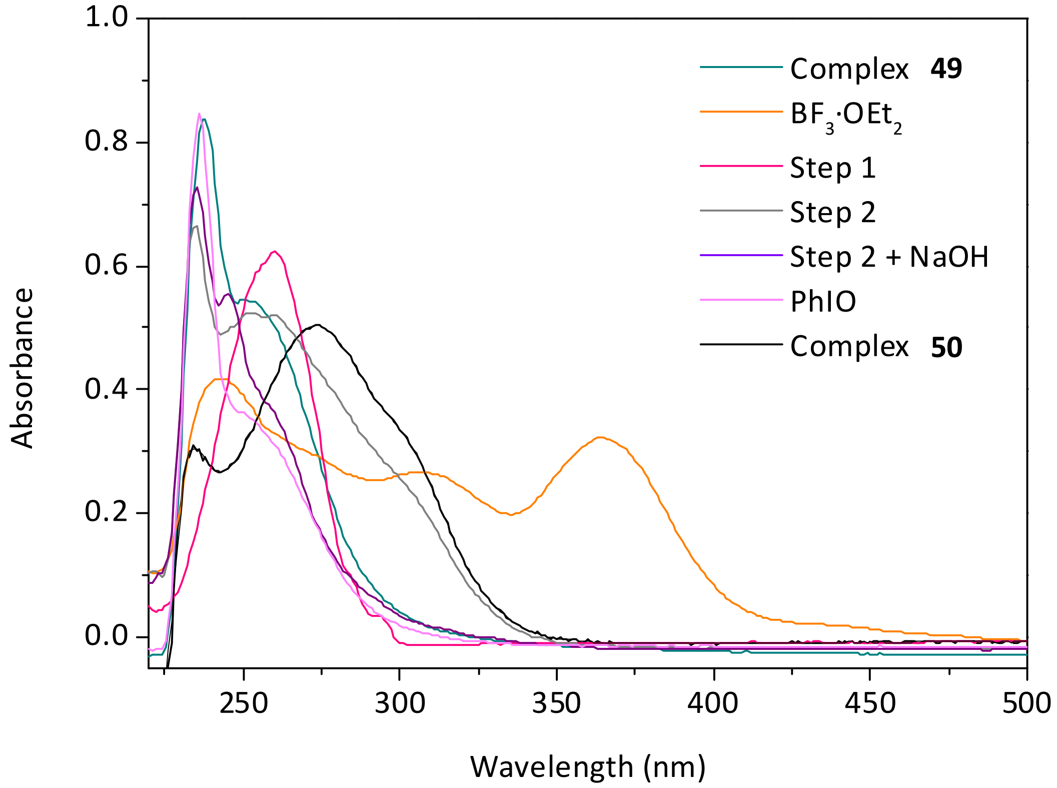

Figure S15. In-situ ultraviolet visible (UV-vis) spectra during the one-pot dehydrogenative silylation of styrene **5** with $HSiEt_3$ and oxidative dehydrosilylation reaction with the equimolar mixture of $BF_3·OEt_2$ and PhIO. For the sake of comparison, the spectrum of the isolated compound **50** is also included.

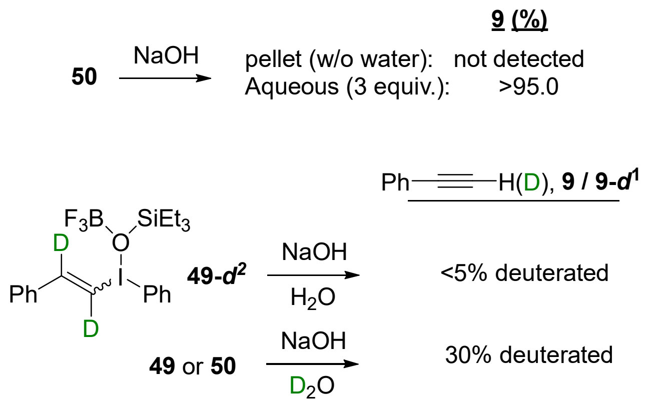

Figure S16. Isotopic reactive evidences for the proposed reaction mechanism.

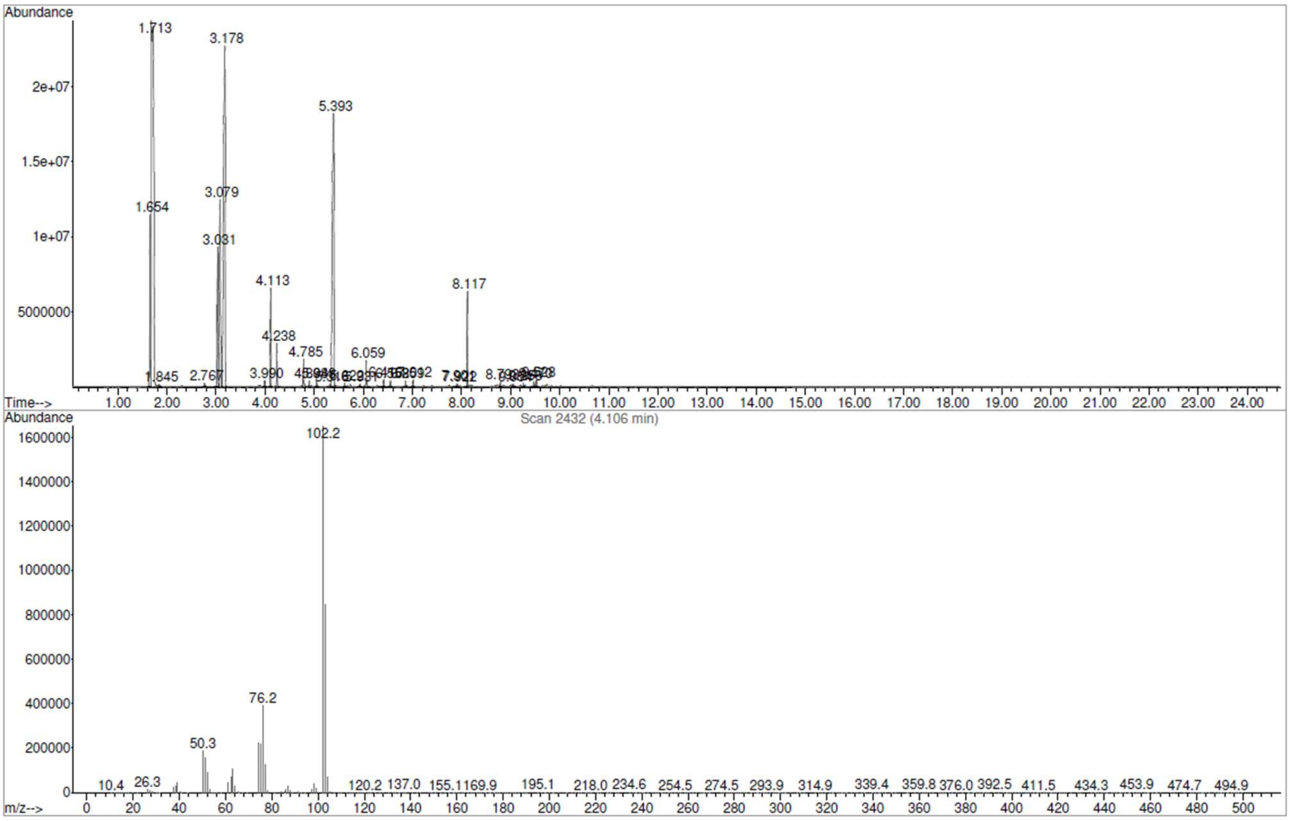

Figure S17. GC-MS spectrum of isolated compound **50** after treatment with $NaOH_{aq.}$ (3 equivalents) in D_2O for 1 h at room temperature. The spectrum coincides with a mixture of phenylacetylene **9** and **9-*d*^{1}** (66.6% according to the relative intensity of the 102 / 103 Da. peaks).

Tables.

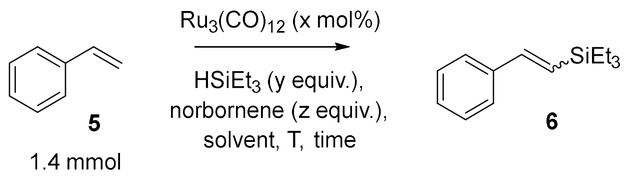

Table S1. Optimization of the dehydrogenative silylation reaction catalyzed by $Ru_3(CO)_{12}$. The *trans* to *cis* ratio for **6** is typically >10:1. GC results. Each experiment was reproduced at least once.

Entry	$HSiEt_3$ (mmol)	$Ru_3(CO)_{12}$ (mol%)	Toluene (mL)	Norbornene (mmol)	Temperature (ºC)	Time (min)	Yield to 6 (%)
**1**	2	0.75	2	2.97	80	30	>99.0
**2**	2	0.5	2	2.93	80	45	>99.0
**3**	2	0.3	2	3.02	80	45	>99.0
**4**	1.7	0.5	2	2.3	80	90	>99.0
**5**	1.7	0.5	2	1.8	80	90	96.3
**6**	1.7	0.5	CH_2Cl_2	2.3	40	1440 (24 h)	0.0
**7**	1.7	0.5	CH_2Cl_2	2.3	60	1440 (24 h)	31.1
**8**	1.7	0.5	CH_2Cl_2	2.3	80	120	85.6
**9**	**1.7**	**0.5**	**1**	**2.3**	**80**	**30**	**>99.0**
**10**	1.7	0.5	1,2-Dichlorobenzene	2.3	80	60	98.2
**11**	1.7	0.5	THF	2.3	80	60	0.0
**12**	1.7	0.5	Acetonitrile	2.3	80	60	0.0
**13**	1.7	0.5	Chloroform	2.3	80	60	43.6
**14**	1.7	0.5	1	2.3	60	1440 (24 h)	86.1
**15**	1.7	0.5	1	2.3	70	120	>99.0
**16**	1.7	0.2	1	2.3	80	94	62.8
**17**	1.7	0.3	1	2.3	80	120	95.0
**18**	1.7	0.4	1	2.3	80	90	>99.0

Table S2. Results for the olefin metathesis reaction between **5** and **6´** catalyzed by ruthenium complexes, under the reaction conditions described in Figure S5. GC results.

**Entry**	**Catalyst**	**Yield of 3 (%),[a] DCM, 20 ºC**	**Yield of 3 (%),[a] Toluene, 70 ºC**
**1**	LatMet	8.4 (0)	-
**2**	AquaMet	44.6 (0)	39.2 (0)
**3**	FixCa	26.1 (0)	-
**4**	HeatMet	22.3 (0)	-
**5**	Nitro-Grela	58.2 (0)	34.1 (0)
**6**	GreenCat	87.8 (0)	-
**7**	StickyCat	43.6 (0)	39.2 (0)
**8**	Grubbs 2nd gen	44.8 (0)	-
**9**	Hoveyda-Grubbs	26.1 (0)	-

[a] Between parentheses, yield of the desired product **6´**. Product **3** was obtained as a mixture of *cis* and *trans*-stilbene **SI-8**.

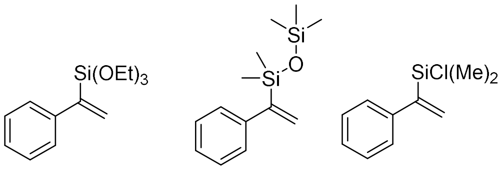

SI-12 **SI-13** **SI-14**

Table S3. Results for the synthesis of other α-vinylsilanes to be used as reagents in the olefinic metathesis reaction (following the procedure in Ref. 31 in the main text).

**Entry**	**Reagent**	**Conversion of 9 (%)**	**Selectivity (α/β)**	**Yield of products SI-12/SI-14**
**1**	Triethoxysilane	72.9	100/0	72.9 (**S12**)
**2**	Pentamethyldisiloxane	97.8	50/50	48.9 (**S13**)
**3**	Chlorodimethylsilane	89.5	0/100	0 (**S14**)

Table S4. Catalytic results for the metathesis reaction between styrene **5**, 1-pentene or 1-hexene **1** with **S12,** using GreenCat or the Schrock's catalyst, under the reaction conditions described in Figure S5.

Entrance	Reagent	Catalyst	Conversion alkene, %	Selectivity to the alkene/vinyl silane metathesis product, %
**1**	**5**	GreenCat	39.7	0
**2**	1-pentene	GreenCat	0	0
**3**	**5**	Schrock's	67.0	0
**4**	**1**	Schrock's	68.6	0

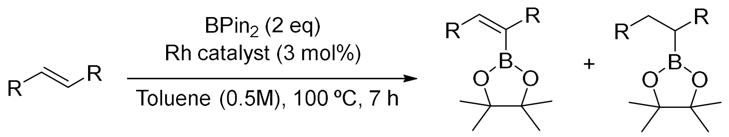

Table S5. Catalytic results for the dehydrogenative monoborylation reaction, following the reaction conditions in Ref. 30 in the main text.

Entry	Reagent	Conversion, %	Selectivity to alkene, %	Selectivity to alkane, %
**1**	*cis*-Stilbene **SI-8**	90.6	0	>99.0
**2**	Cyclododecene **SI-9**	93.3	>99.0	0

Characterization of isolated compounds.

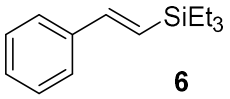

6

(*E*)-triethyl(styryl)silane (6). GC-MS (m/z, $M^{+\bullet}$ 218, major peaks found: 218, 189, 160, 131, 103, 91, 77. ^{1}H NMR (401 MHz, $CDCl_3$) δ 7.37 (d, J = 7.9 Hz, 2H), 7.25 (t, J = 7.9 Hz, 2H), 7.16 (t, J = 7.9 Hz, 1H), 6.82 (d, J = 19.3 Hz, 1H), 6.35 (d, J = 19.3 Hz, 1H), 0.91 (t, J = 8.0 Hz, 9H), 0.59 (q, J = 8.0 Hz, 6H). ^{13}C NMR (101 MHz, $CDCl_3$) δ 145.0 (CH), 138.7 (CH_2), 128.6 (CH), 128.0 (CH), 126.5 (CH), 126.1 (CH), 7.5 (CH_3), 3.7 (CH_2). IR: ν = 2952, 2910, 2872, 1601, 1575, 1494, 1457, 1446, 1414, 1378, 1332, 1289, 1236, 1214, 1196, 1010, 1069, 988, 912, 827, 784, 734, 720, 687, 596, 555, 487, 421 cm^{-1}.

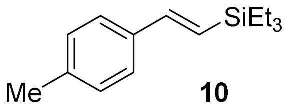

10

(*E*)-triethyl(4-methylstyryl)silane (10). GC-MS (m/z, $M^{+\bullet}$ 232), major peaks found: 232, 203, 175, 145, 119, 105, 87, 59. ^{1}H NMR (401 MHz, $CDCl_3$) δ 7.37 (d, J = 8.0 Hz, 2H), 7.16 (d, J = 8.0 Hz, 2H), 6.90 (d, J = 19.3 Hz, 1H), 6.39 (d, J = 19.3 Hz, 1H), 2.37 (s, 3H), 1.02 (t, J = 8.0 Hz, 9H), 0.69 (q, J = 8.0 Hz, 6H). ^{13}C NMR (101 MHz, $CDCl_3$): δ 144.9 (CH), 137.9 (C), 136.0 (C), 129.3 (CH), 126.4 (CH), 124.7 (CH), 21.3 (CH_3), 7.6 (CH_3), 3.7 (CH_3). IR: ν = 2950, 2909, 2873, 2008, 1606, 1566, 1509, 1456, 1413, 1377, 1329, 1286, 1236, 1196, 1177, 1115, 1015, 986, 843, 789, 778, 753, 714, 617, 591, 578, 495, 422 cm^{-1}.

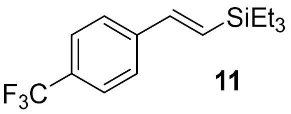

11

(*E*)-triethyl(4-(trifluoromethyl)styryl)silane (11). GC-MS (m/z, $M^{+\bullet}$ 286), major peaks found: 286, 257, 229, 201, 175, 151, 133, 115, 87, 59. ^{1}H NMR (401 MHz, $CDCl_3$) δ 7.58 (d, J = 8.4 Hz, 2H), 7.53 (d, J = 8.4 Hz, 2H), 6.92 (d, J = 19.3 Hz, 1H), 6.56 (d, J = 19.3 Hz, 1H), 1.00 (t, J = 8.0 Hz, 9H), 0.68 (q, J = 8.0 Hz, 6H). ^{13}C NMR (101 MHz, $CDCl_3$) δ 143.4 (CH), 141.9 (C), 129.9 (CH), 129.6 (C), 126.1 (CH), 125.6 (CH), 123.0 (C), 7.5 (CH_3), 3.6 (CH_2). IR: ν = 2954, 2911, 2875, 1917, 1614, 1573, 1457, 1412, 1379, 1321, 1237, 1213, 1201, 1163, 1122, 1105, 1066, 1015, 986, 953, 857, 836, 793, 785, 754, 719, 689, 665, 625, 592, 501, 445 cm^{-1}.

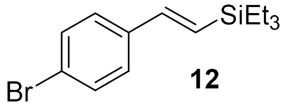

12

(*E*)-(4-bromostyryl)triethylsilane (12). GC-MS (m/z, $M^{+\bullet}$ 296), major peaks found: 296, 269, 239, 211, 185, 131, 105, 87, 59. ^{1}H NMR (401 MHz, $CDCl_3$) δ 7.45 (d, J = 8.5 Hz, 2H), 7.30 (d, J = 8.5 Hz, 2H), 6.81 (d, J = 19.3 Hz, 1H), 6.42 (d, J = 19.3 Hz, 1H), 0.98 (t, J = 7.9 Hz, 9H), 0.64 (q, J = 7.9 Hz, 6H). ^{13}C NMR (101 MHz, $CDCl_3$) δ 143.6 (CH), 137.6 (C), 131.7 (CH), 128.0 (CH), 127.3 (CH), 121.8 (C), 7.5 (CH_3), 3.6 (CH_2). IR: ν = 2951, 2908, 2872, 1895, 1771, 1630, 1601, 1587, 1559, 1484, 1456, 1413, 1395, 1376, 1325, 1278, 1236, 1211, 1195, 1175, 1106, 1072, 1007, 985, 911, 845, 830, 780, 717, 634, 580, 573, 492, 456, 434 cm^{-1}.

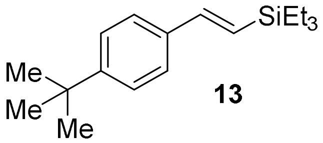

13

(*E*)-(4-(tert-butyl)styryl)triethylsilane (13). GC-MS (m/z, $M^{+\bullet}$ 274), major peaks found: 274, 245, 217, 189, 161, 145, 131, 115, 101, 87, 57, 41, 29. ^{1}H NMR (401 MHz, $CDCl_3$) δ 7.39 (d, *J* = 8.5 Hz, 2H), 7.36 (d, *J* = 8.5 Hz, 2H), 6.88 (d, *J* = 19.3 Hz, 1H), 6.38 (d, *J* = 19.3 Hz, 1H), 1.32 (s, 9H), 0.98 (t, *J* = 8.0 Hz, 9H), 0.65 (q, *J* = 8.0 Hz, 6H). ^{13}C NMR (101 MHz, $CDCl_3$): δ 151.2 (C), 144.7 (CH), 136.0 (C), 126.2 (CH), 125.6 (CH), 125.0 (CH), 34.7 (C), 31.5 (CH), 7.6 (CH_3), 3.7 (CH_2). IR: ν = 2953, 2907, 2873, 2009, 1604, 1558, 1511, 1491, 1461, 1456, 1410, 1394, 1375, 1362, 1328, 1289, 1268, 1236, 1204, 1184, 1110, 1013, 986, 943, 848, 822, 785, 721, 665, 616, 588, 577, 549, 468, 420 cm^{-1}.

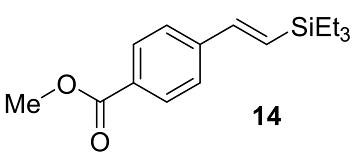

14

Methyl (*E*)-4-(2-(triethylsilyl)vinyl)benzoate (14). GC-MS (m/z, $M^{+\bullet}$ 170), major peaks found: : 275, 247, 219, 189, 161, 145, 129, 115, 105, 94, 80, 59. ^{1}H NMR (401 MHz, $CDCl_3$) δ 7.92 (d, J = 8.4 Hz, 2H), 7.42 (d, J = 8.4 Hz, 2H), 6.85 (d, J = 19.3 Hz, 1H), 6.50 (d, J = 19.3 Hz, 1H), 3.83 (s, 3H), 0.92 (t, J = 7.9 Hz, 3H), 0.60 (q, J = 7.9 Hz, 6H). ^{13}C NMR (101 MHz, $CDCl_3$) δ 167.1 (C), 143.9 (CH), 142.8 (C), 130.0 (CH), 130.0 (CH), 126.3 (CH), 52.2 (CH_3), 7.5 (CH_3), 3.6 (CH_2). IR: ν = 2951, 2909, 2875, 1725, 1606, 1559, 1541, 1521, 1507, 1457, 1434, 1416, 1408, 1377, 1309, 1277, 1240, 1214, 1195, 1176, 1106, 1016, 987, 972, 914, 868, 842, 807, 783, 754, 733, 723, 690 cm^{-1}.

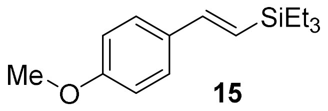

15

(*E*)-triethyl(4-methoxystyryl)silane (15). GC-MS (m/z, $M^{+\bullet}$ 248), major peaks found: 248, 219, 191, 163, 147, 137, 119, 105, 95, 81 59. ^{1}H NMR (401 MHz, $CDCl_3$) δ 7.39 (d, J = 8.8 Hz, 2H), 6.87 (d, J = 8.8 Hz, 2H), 6.85 (d, J = 19.3 Hz, 1H), 6.25 (d, J = 19.3 Hz, 1H), 3.82 (s, 1H), 0.99 (t, J = 7.9 Hz, 9H), 0.65 (q, J = 7.9 Hz, 6H). ^{13}C NMR (101 MHz, $CDCl_3$): δ 159.7 (C), 144.3 (CH), 131.7 (C), 127.7 (CH), 123.2 (CH), 114.0 (CH), 55.46 (CH_3), 7.57 (CH_3), 3.73 (CH_2). IR: ν = 2950, 2907, 2873, 2833, 1922, 1604, 1569, 1508, 1462, 1456, 1439, 1415, 1375, 1331, 1303, 1294, 1249, 1195, 1169, 1105, 1035, 1011, 985, 842, 786, 748, 714, 665, 637, 592, 579, 543, 534, 516, 415 cm^{-1}.

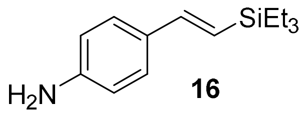

16

(*E*)-4-(2-(triethylsilyl)vinyl)aniline (16). GC-MS (m/z, $M^{+\bullet}$ 233, major peaks found: 233, 204, 176, 148, 122, 106, 88, 74, 59. ^{1}H NMR (401 MHz, $CDCl_3$) δ 7.18 (d, J = 8.5 Hz, 2H), 6.71 (d, J = 19.3 Hz, 1H), 6.54 (d, J = 8.5 Hz, 2H), 6.09 (d, J = 19.3 Hz, 1H), 3.63 (s, 2H), 0.90 (t, J = 7.9 Hz, 9H), 0.56 (q, J = 7.9 Hz, 6H). ^{13}C NMR (101 MHz, $CDCl_3$) δ 146.4 (C), 144.7 (CH), 129.8 (C), 127.7 (CH), 121.0 (CH), 115.2 (CH), 7.5 (CH_3), 3.8 (CH_2). IR: ν = 3358, 3212, 2952, 2908, 2874, 1619, 1603, 1569, 1556, 1539, 1511, 1462, 1456, 1432, 1415, 1376, 1330, 1286, 1237, 1197, 1173, 1127, 1012, 985, 840, 792, 772, 729, 719, 592, 511, 433, 418 cm^{-1}.

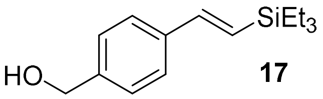

17

(*E*)-(4-(2-(triethylsilyl)vinyl)phenyl)methanol (17). GC-MS (m/z, $M^{+\bullet}$ 248), major peaks found: 248, 219, 189, 163, 149, 132, 117, 103, 91, 59.

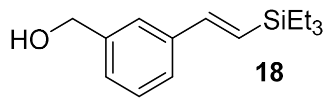

18

(*E*)-(3-(2-(triethylsilyl)vinyl)phenyl)methanol (18). GC-MS (m/z, $M^{+\bullet}$ 246, major peaks found: 246, 217, 189, 159, 131, 115, 105, 94, 80, 59. ^{1}H NMR (401 MHz, $CDCl_3$) δ 7.54 - 7.15 (m, 4H), 6.90 (d, J = 19.3 Hz, 1H), 6.43 (d, J = 19.3 Hz, 1H), 4.74 (d, J = 1.4 Hz, 2H), 0.99 (t, J = 7.9 Hz, 9H), 0.67 (q, J = 7.9 Hz, 6H). ^{13}C NMR (101 MHz, $CDCl_3$) δ 145.0 (CH), 141.7 (C) , 137.7 (C), 128.6 (CH), 126.0 (CH), 124.2 (CH), 64.8 (CH_2), 7.4 (CH_3), 3.7 (CH_2). IR: ν = 2952, 2908, 2873, 1605, 1581, 1457, 1414, 1374, 1238, 1200, 1155, 1102, 1075, 1005, 987, 971, 904, 888, 814, 792, 766, 741, 721, 688, 601, 557, 522, 419 cm^{-1}.

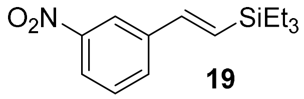

19

(E)-triethyl(3-nitrostyryl)silane (19). GC-MS (m/z, $M^{+\bullet}$ 262), major peaks found: 262, 234, 206, 178, 161, 146, 131, 115, 105, 87, 59.

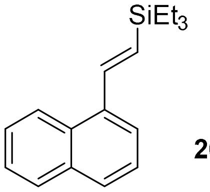

20

(*E*)-triethyl(2-(naphthalen-1-yl)vinyl)silane (20). GC-MS (m/z, $M^{+\bullet}$ 268, major peaks found: 268, 239, 211, 181, 165, 152, 129, 115, 101, 87, 73, 59. ^{1}H NMR (401 MHz, $CDCl_3$) δ 8.12 - 7.30 (m, 8H), 6.42 (d, J = 19.1 Hz, 1H), 0.98 (t, J = 7.9 Hz, 9H), 0.66 (q, J = 7.9 Hz, 1H). ^{13}C NMR (75 MHz, $CDCl_3$) δ 146.5 (C), 142.2 (CH), 136.9 (C), 133.6 (C), 130.9 (CH), 130.3 (CH), 128.5 (CH), 128.0 (CH), 126.0 (CH), 125.7 (CH), 125.6 (CH), 123.6 (CH), 7.5 (CH_3), 3.6 (CH_2). IR: ν = 3058, 2952, 2909, 2872, 1599, 1508, 1456, 1416, 1391, 1376, 1348, 1262, 1235, 1217, 1195, 1014, 986, 905, 805, 786, 769, 727, 648, 608, 566, 539, 445, 420 cm^{-1}.

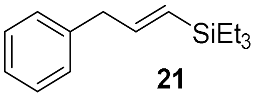

21

(*E*)-triethyl(3-phenylprop-1-en-1-yl)silane (21). GC-MS (m/z, $M^{+\bullet}$ 232, major peaks found: 232, 203, 157, 147, 115, 91, 59. ^{1}H NMR (401 MHz, $CDCl_3$) δ 7.49 - 7.29 (m, 5H), 6.90 (d, J = 19.3 Hz, 1H), 6.45 (d, J = 19.3 Hz, 1H), 4.71 (s, 2H), 0.98 (t, J = 7.9 Hz, 9H), 0.66 (q, J = 7.9 Hz, 6H). ^{13}C NMR (101 MHz, $CDCl_3$) δ 144.7 (CH), 141.2 (C), 139.0 (CH), 128.9 (CH), 126.7 (CH), 126.6 (CH), 125.9 (CH), 124.9 (CH),

65.5 (CH_2), 7.54 (CH_3), 3.65 (CH_2). IR: ν = 3024, 2951, 2910, 2873, 1644, 1616, 1601, 1494, 1455, 1415, 1377, 1238, 1145, 1072, 1016, 1003, 993, 960, 909, 840, 766, 727, 693, 563, 500 cm^{-1}.

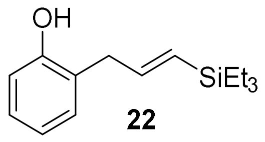

22

(*E*)-2-(3-(triethylsilyl)allyl)phenol (22). GC-MS (m/z, $M^{+\bullet}$ 248), major peaks found: 248, 219, 191, 163, 135, 113, 91, 59, 28.

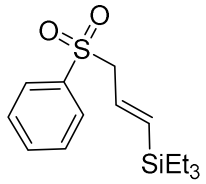

23

Triethyl((phenylsulfonyl)methyl)silane (23). GC-MS (m/z, $M^{+\bullet}$ 280, major peaks found: 280, 253, 227, 189, 163, 135, 115, 103, 87, 59. ^{1}H NMR (300 MHz, $CDCl_3$) δ 7.88 - 7.40 (m, 5H), 5.87 (dt, J = 18.6, 6.9 Hz, 1H), 5.59 (dt, J = 18.6, 1.1 Hz, 1H), 3.81 (dd, J = 6.9, 1.1 Hz, 2H), 0.86 (t, J = 7.9 Hz, 9H), 0.45 (q, J = 7.9 Hz, 6H).

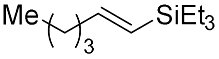

25

(*E*)-triethyl(hex-1-en-1-yl)silane (25). GC-MS (m/z, $M^{+\bullet}$ 198), major peaks found: 198, 169, 141, 113, 85, 59, 27. ^{1}H NMR (401 MHz, $CDCl_3$) δ 5.96 (dt, J = 18.7, 6.3 Hz, 1H), 5.46 (dt, J = 18.7, 1.5 Hz, 1H), 2.05 (dd, J = 13.0, 6.3 Hz, 2H), 1.35 - 1.15 (m, 6H), 0.85 (t, J = 7.9 Hz, 9H), 0.47 (q, J = 7.9 Hz, 6H). ^{13}C NMR (101 MHz, $CDCl_3$) δ 149.0 (CH), 125.7 (CH), 37.0 (CH_2), 31.3 (CH_2), 22.4 (CH_2), 14.1 (CH), 7.5 (CH_3), 3.8 (CH_2). IR: ν = 2954, 2931, 2911, 2874, 2067, 2015, 1617, 1457, 1417, 1376, 1239, 1157, 1014, 990, 967, 907, 782, 730, 719, 650, 582 cm^{-1}.

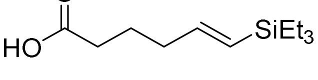

26

(*E*)-6-(triethylsilyl)hex-5-enoic acid (26). GC-MS (m/z, $M^{+\bullet}$ 228), major peaks found: 228, 199, 157, 145, 103, 87, 75, 59, 47.

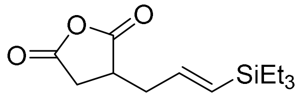 **27**

(*E*)-3-(3-(triethylsilyl)allyl)dihydrofuran-2,5-dione (27). GC-MS (m/z, $M^{+\bullet}$ 254), major peaks found: 254, 225, 197, 153, 125, 103, 75, 43.

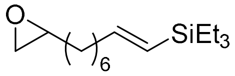 **28**

(*E*)-triethyl(7-(oxiran-2-yl)hept-1-en-1-yl)silane (28). GC-MS (m/z, $M^{+\bullet}$ 268), major peaks found: 268, 239, 211, 181, 153, 115, 87, 59, 31. ^{1}H NMR (401 MHz, $CDCl_3$) δ 6.02 (dt, J = 18.7, 6.3 Hz, 1H), 5.53 (dt, J = 18.7, 1.4 Hz, 1H), 2.95 - 2.85 (m, 1H), 2.75 (dd, J = 4.9, 4.1 Hz, 1H), 2.46 (dd, J = 5.0, 2.7 Hz, 1H), 2.11 (td, J = 7.7, 1.4 Hz, 2H), 1.61 - 1.21 (m, 8H), 0.92 (t, J = 7.9 Hz, 12H), 0.54 (q, J = 7.9 Hz, 8H). ^{13}C NMR (101 MHz, $CDCl_3$) δ 148.8 (CH), 125.8 (CH), 52.5 (CH), 47.3 (CH_2), 37.1 (CH_2), 32.6 (CH_2), 29.1 (CH_2), 26.1 (CH_2), 7.5 (CH_3), 3.7 (CH_2). IR: v = 3042, 2951, 2982, 2872, 2856, 1616, 1457, 1414, 1375, 1260, 1237, 1098, 1014, 991, 967, 913, 834, 784, 718, 661, 587, 573, 437 cm^{-1}.

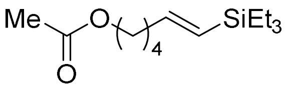 **29**

(*E*)-6-(triethylsilyl)hex-5-en-1-yl acetate (29). GC-MS (m/z, $M^{+\bullet}$ 343), major peaks found: 343, 313, 288, 258, 217, 189, 161, 133, 114, 97, 75, 59, 43.

NMR copies

Vinyl triethyl silane **6**

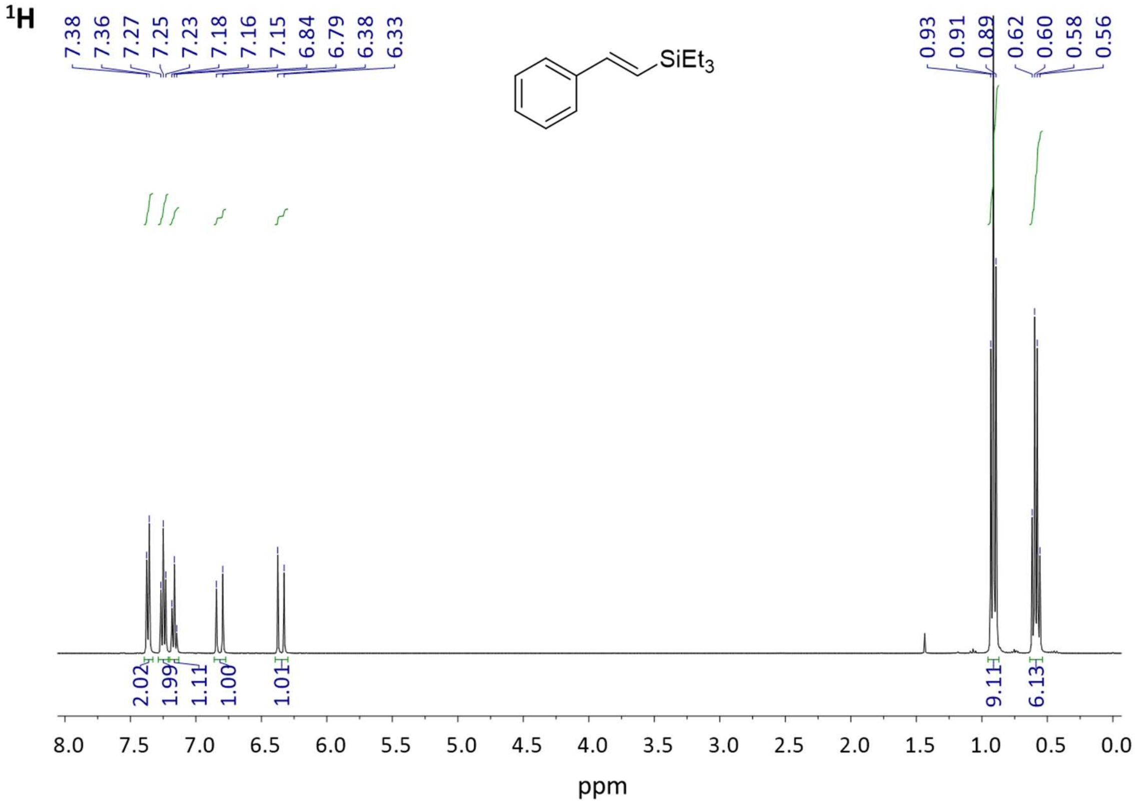

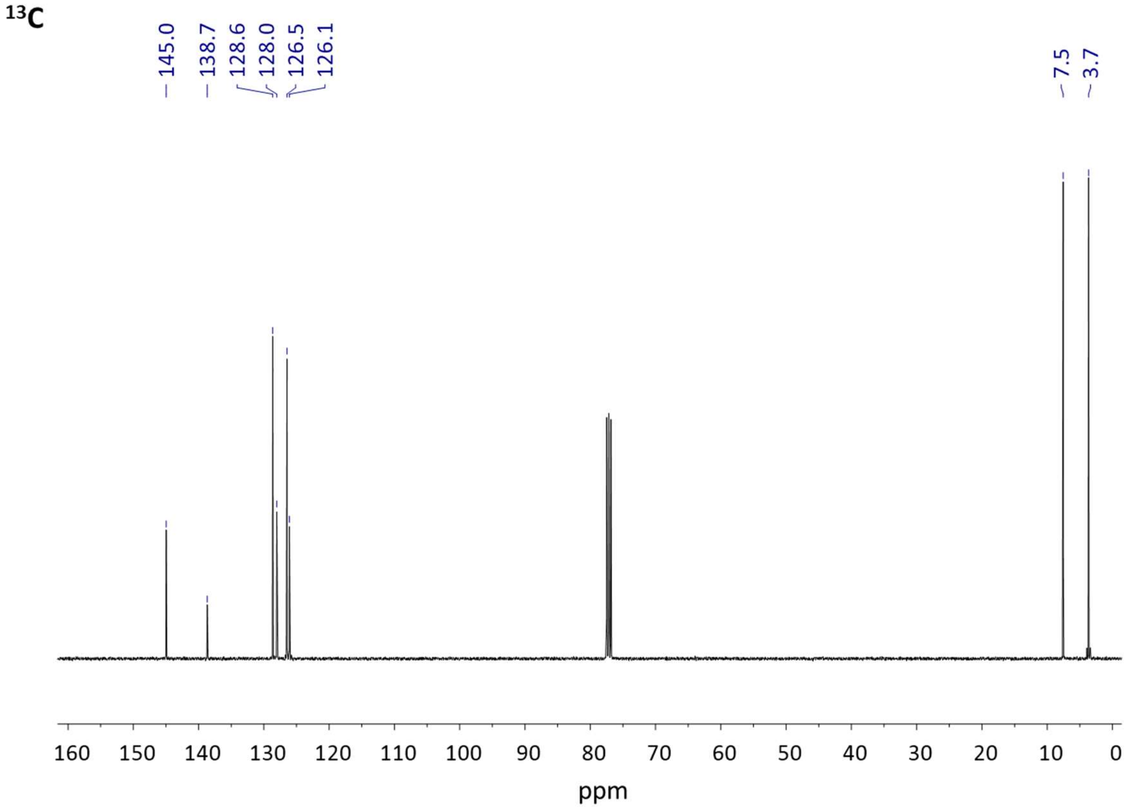

Vinyl triethyl silane **10**

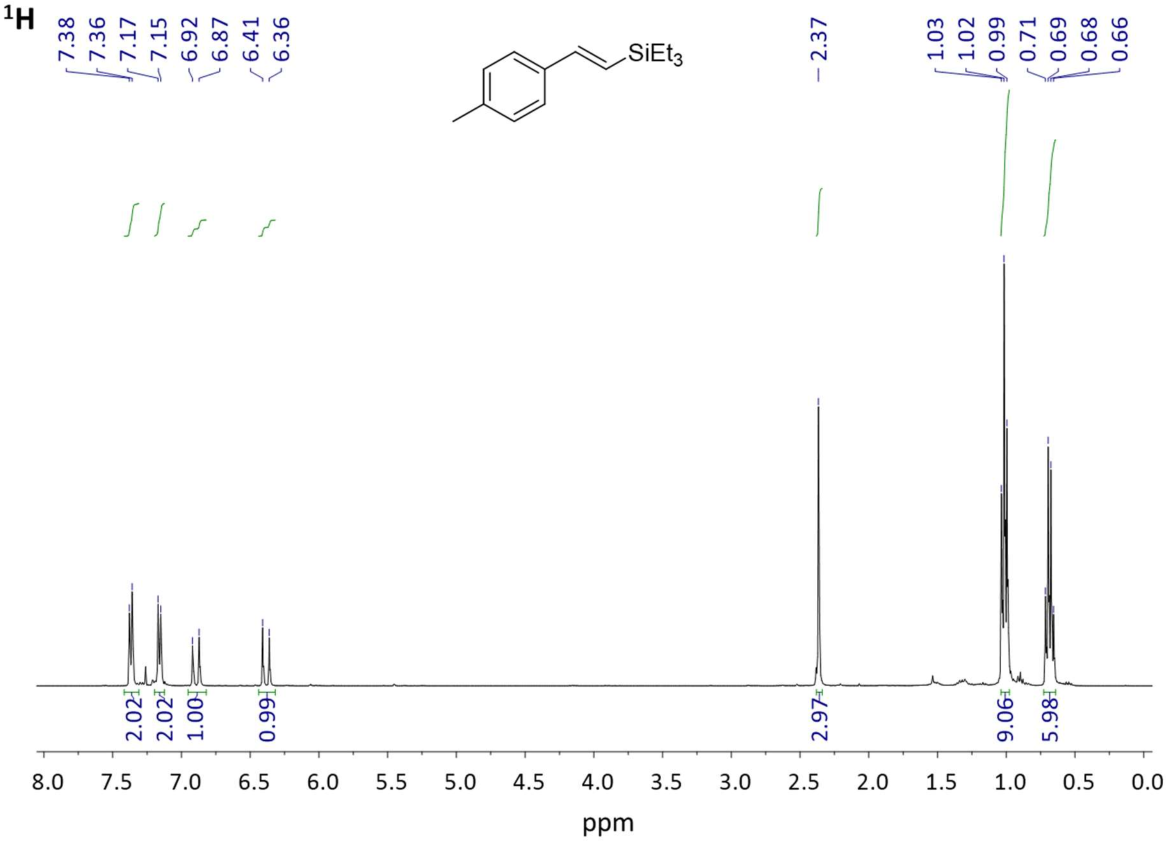

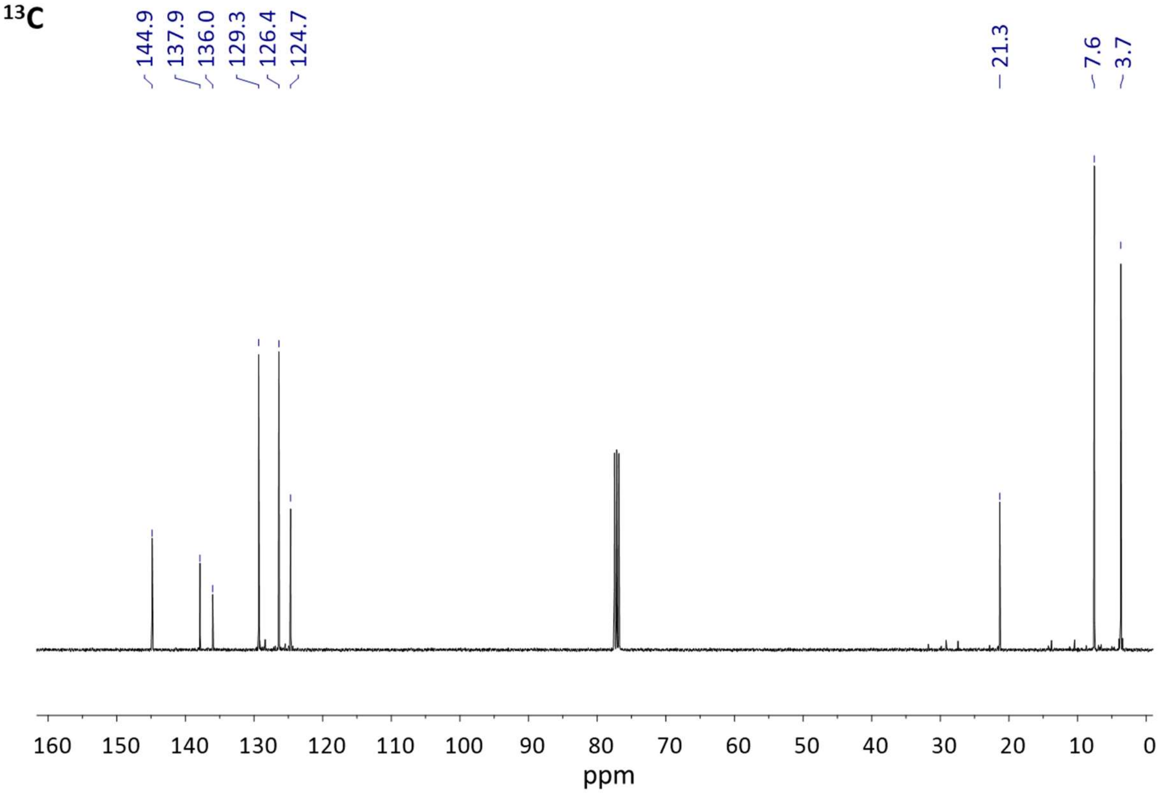

Vinyl triethyl silane **11**

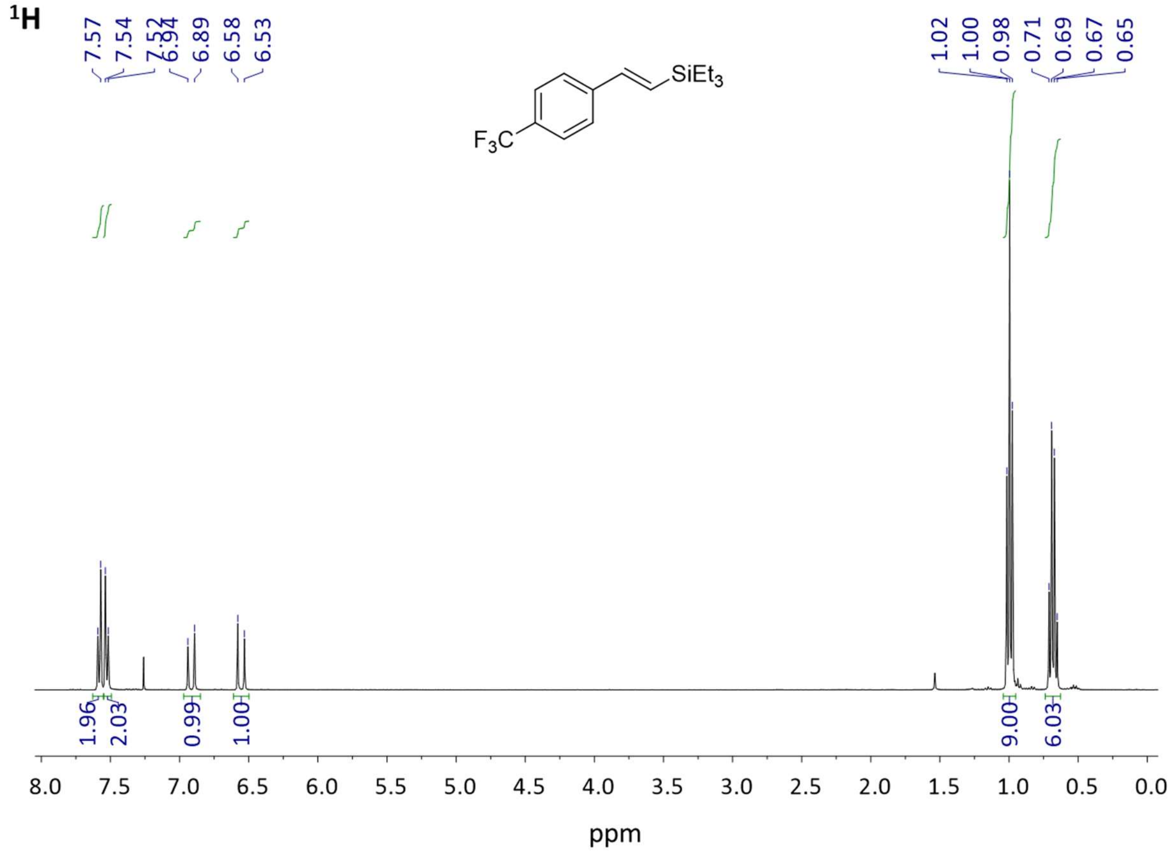

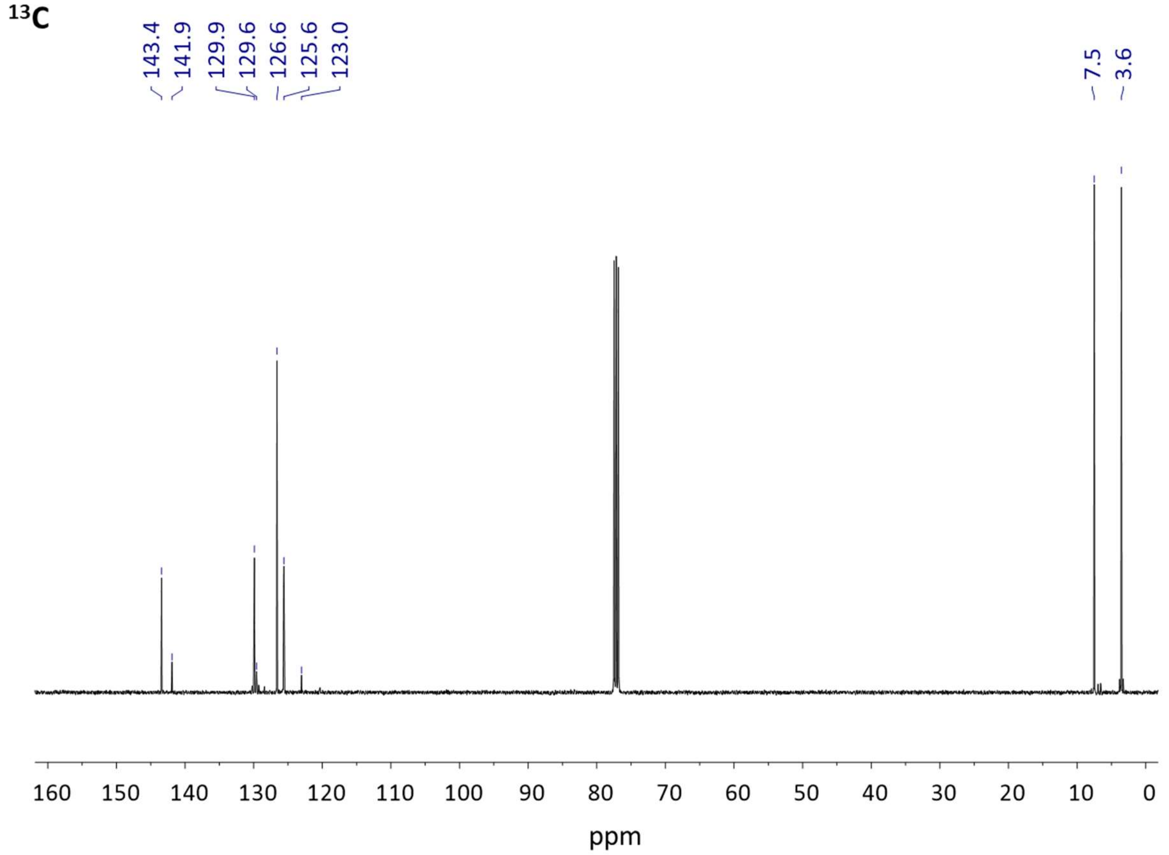

Vinyl triethyl silane **12**

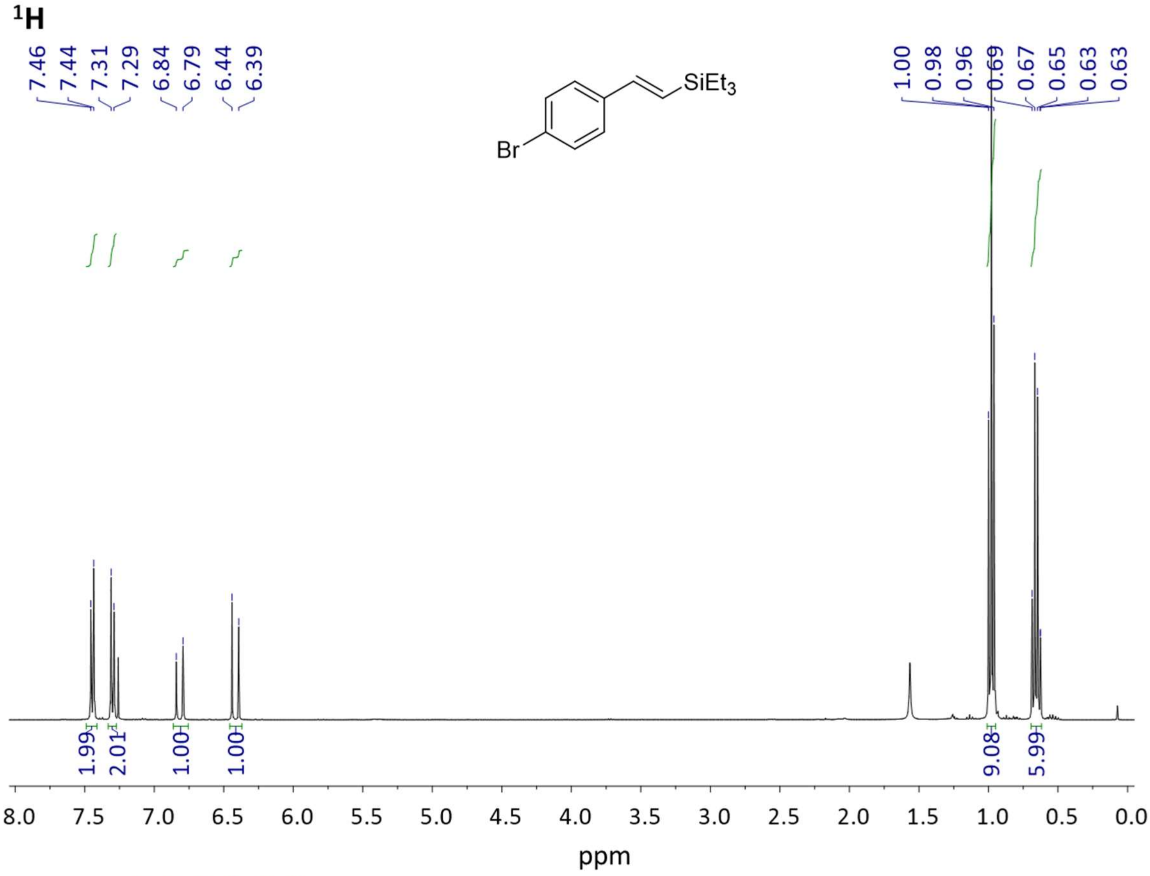

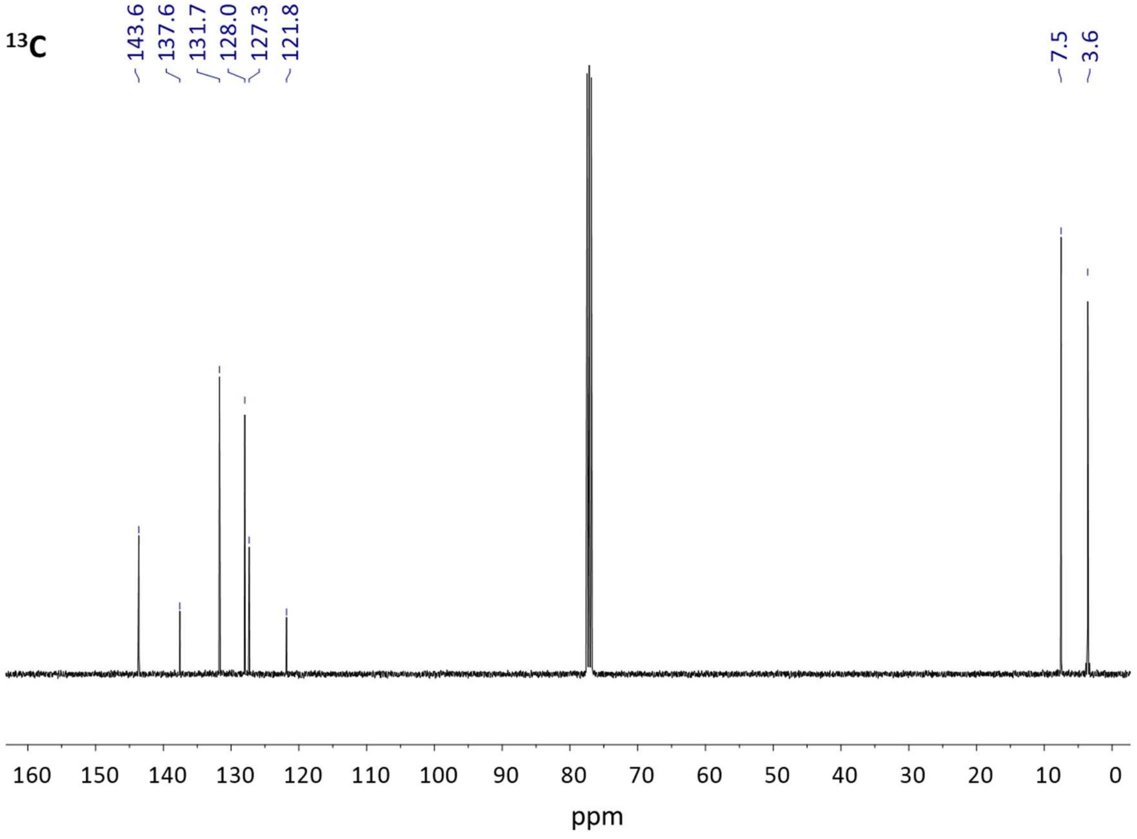

Vinyl triethyl silane **13**

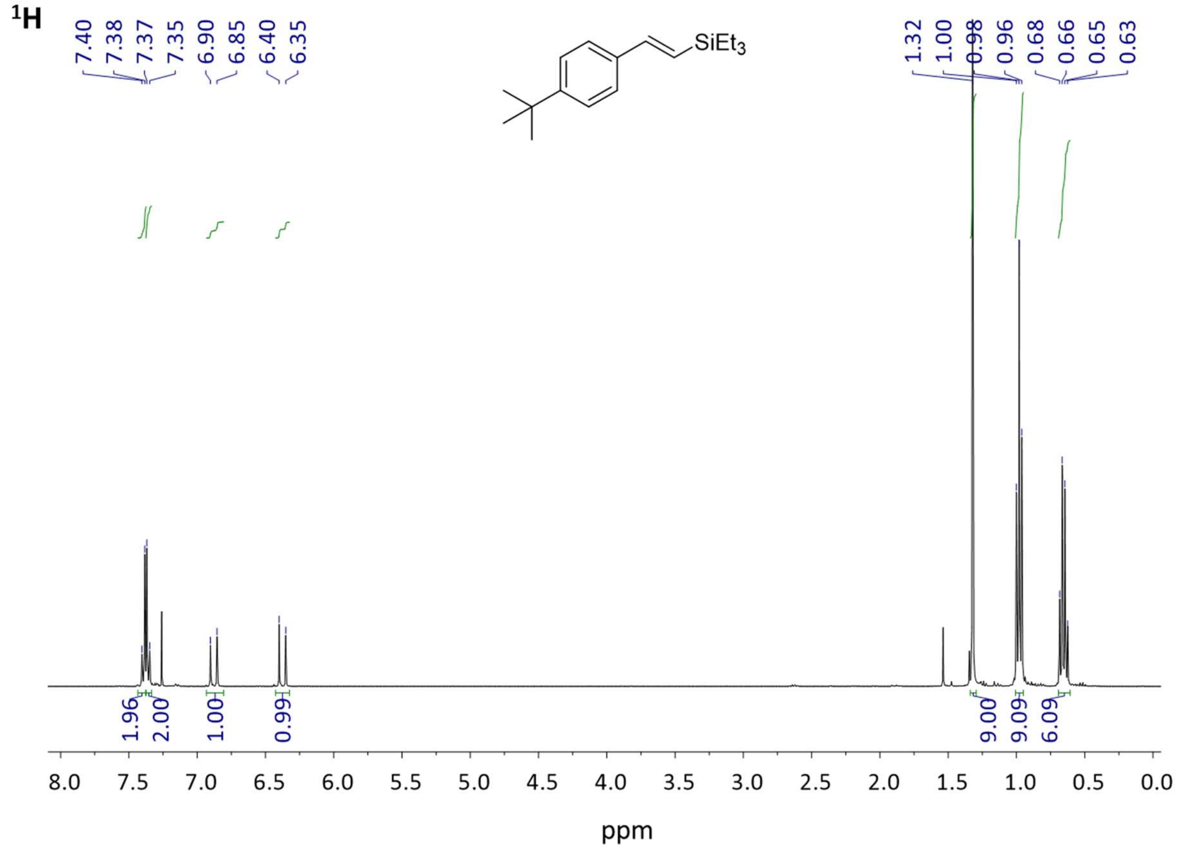

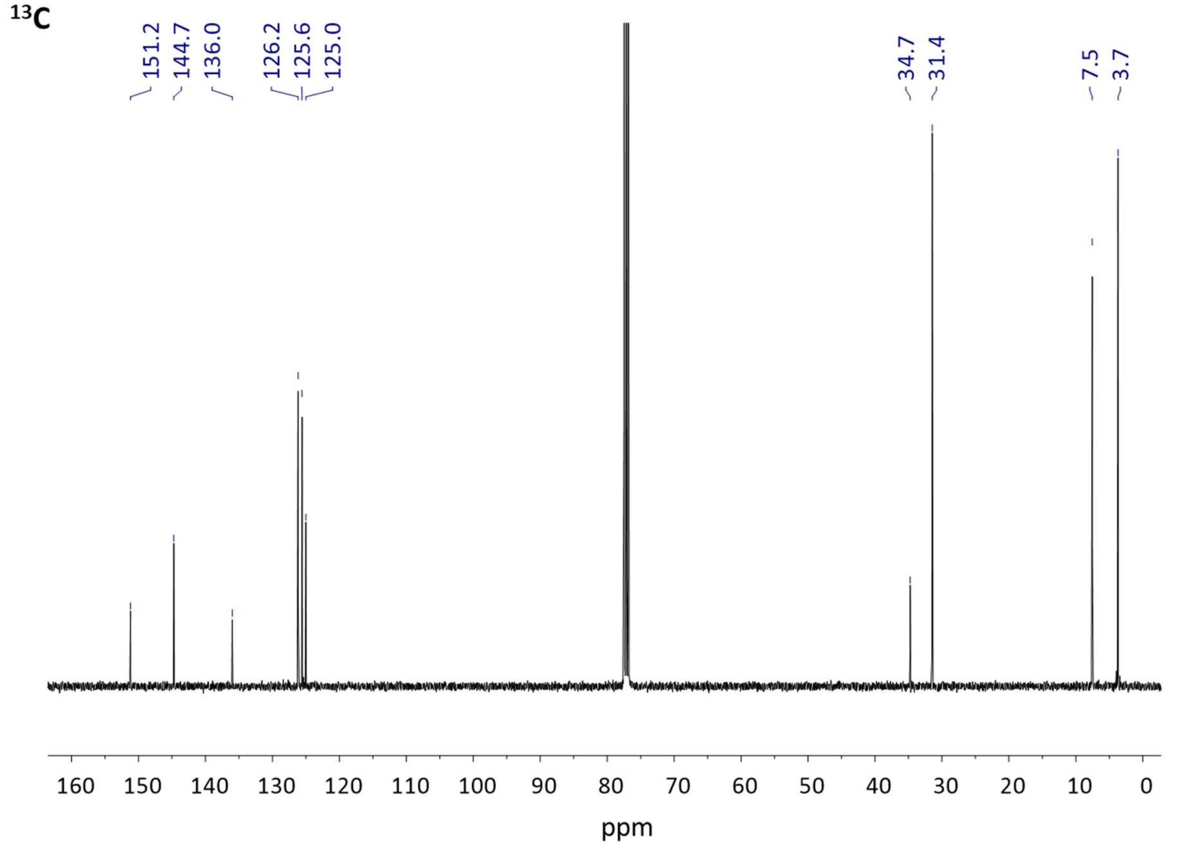

Vinyl triethyl silane **15**

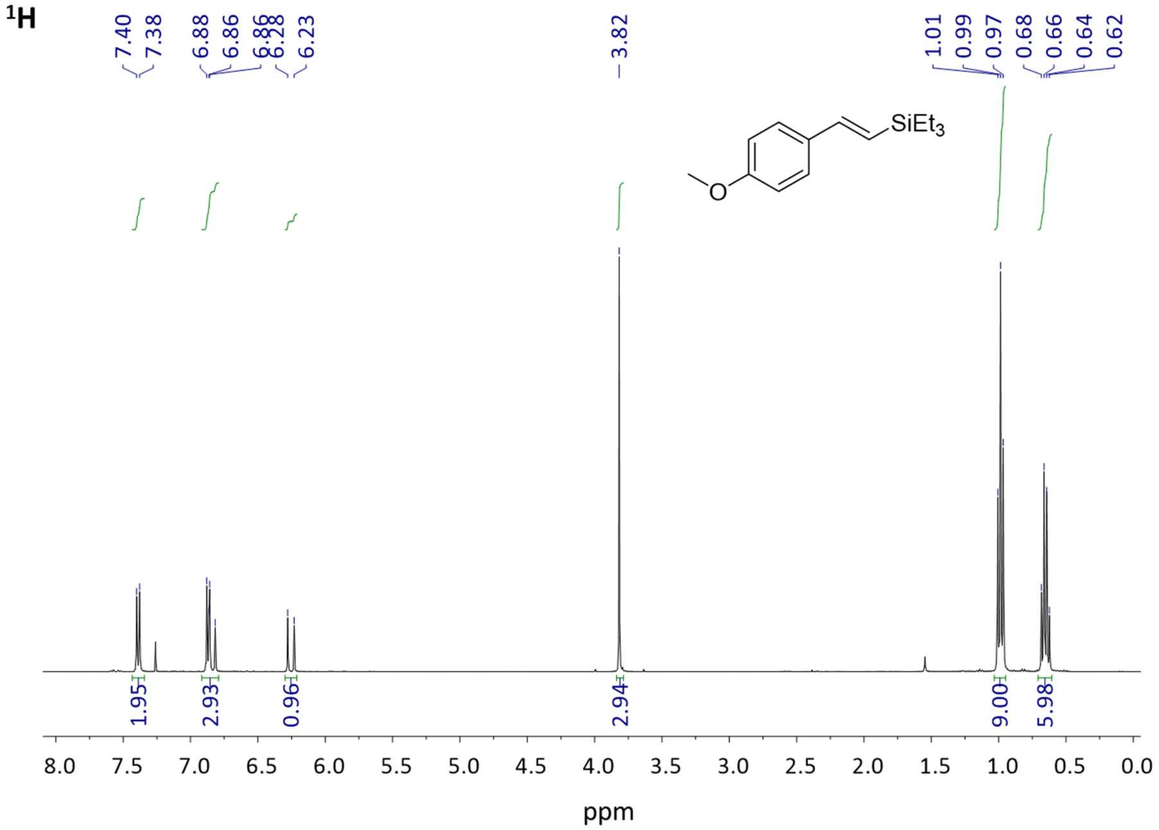

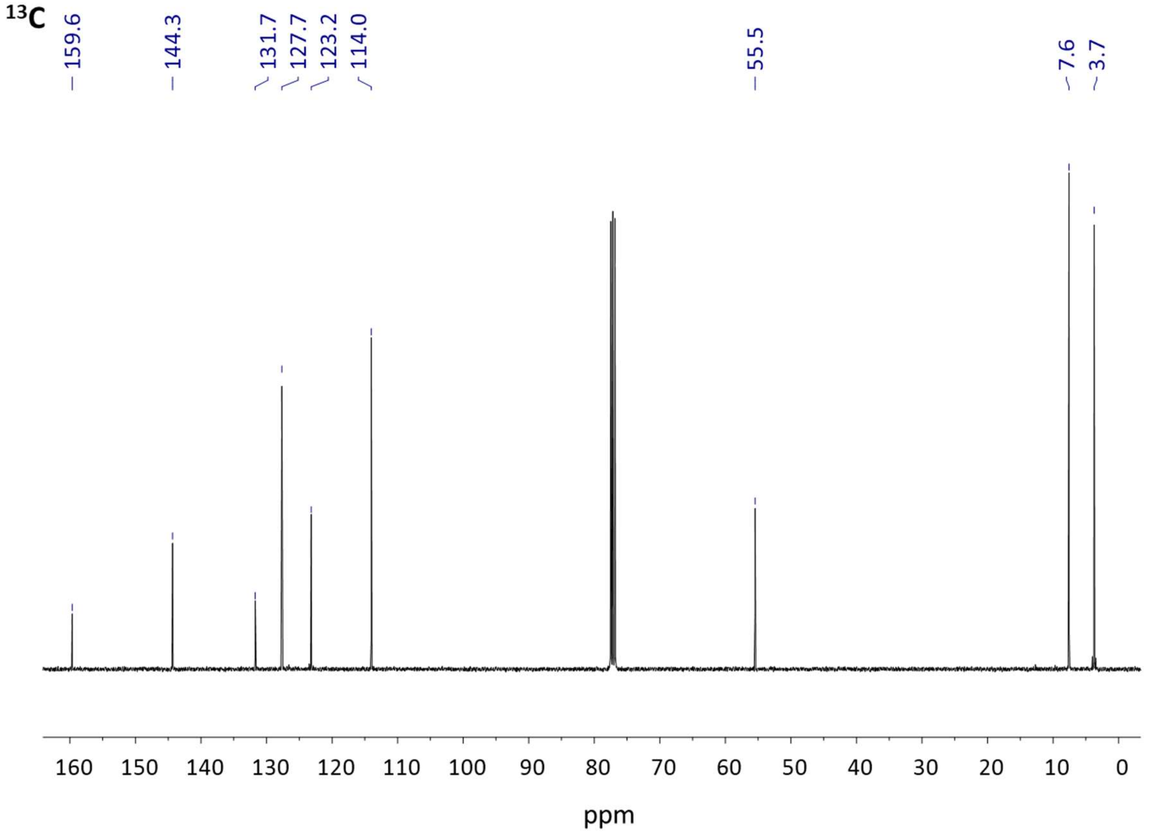

Vinyl triethyl silane **21**

^{1}H

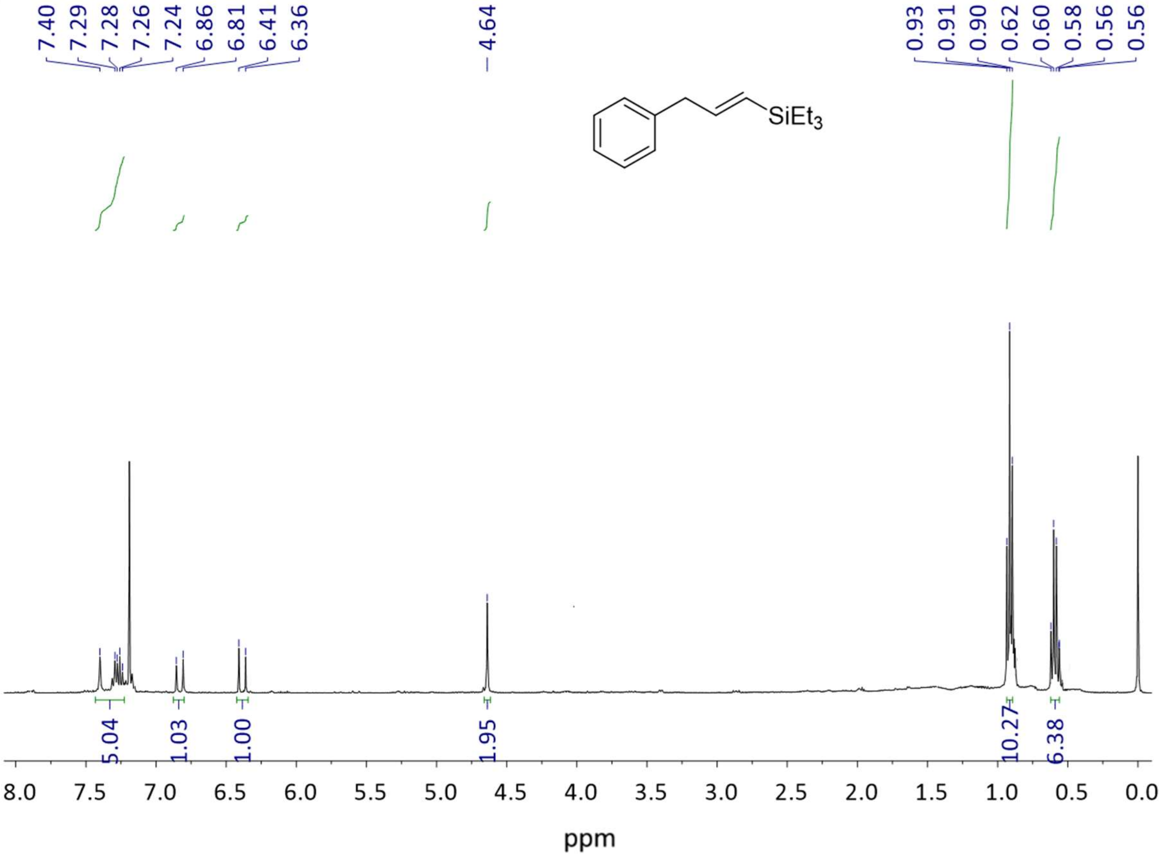

^{13}C

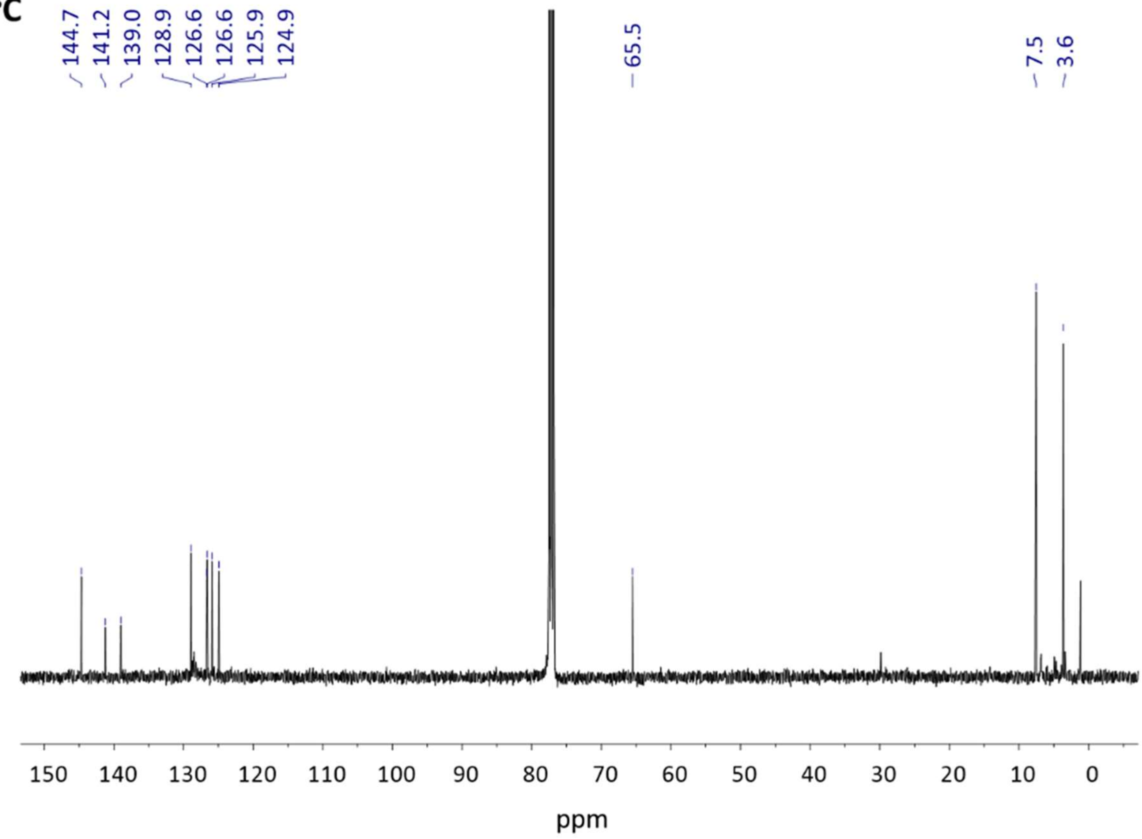

Vinyl triethyl silane **28**

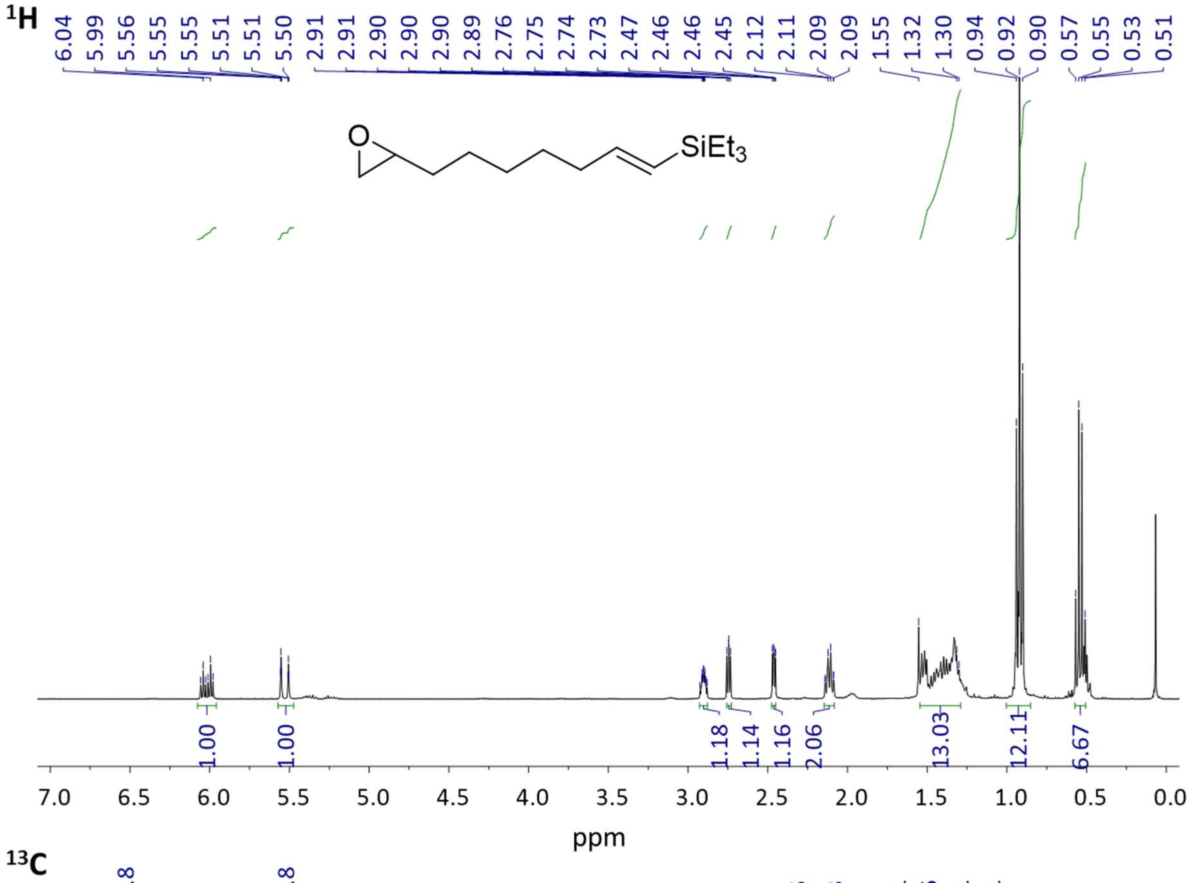

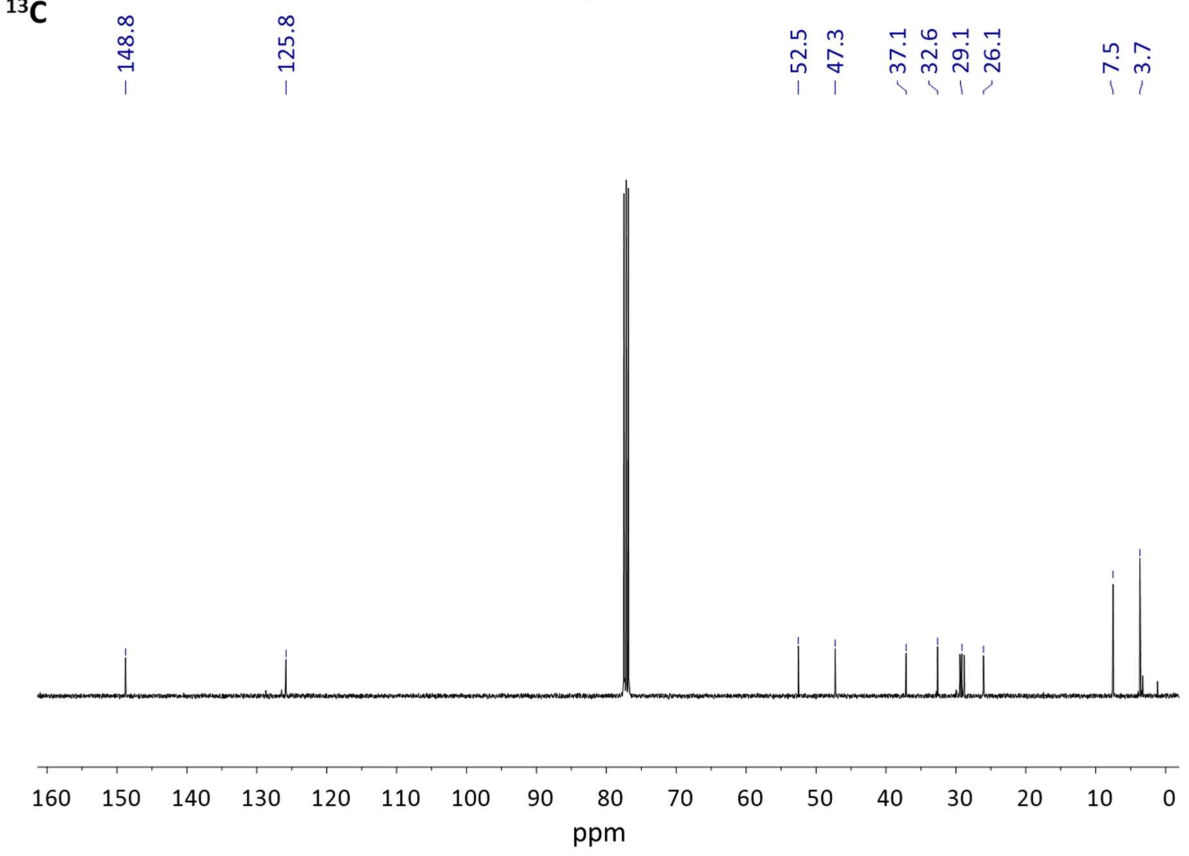

Supplement: Supplementary file 1 — au4c00599_si_001.pdf [file au4c00599_si_001.pdf]
